# Supplementary material for: A systematic evaluation of Mycobacterium tuberculosis Genome-Scale Metabolic Networks
Source: PLoS Comput Biol. 2020 Jun 15;16(6):e1007533. doi: 10.1371/journal.pcbi.1007533 (PMC7316355; doi:10.1371/journal.pcbi.1007533)
Supplement: S1 Appendix — A document providing additional detail about the eight Mtb GSMNs used in this study. (DOCX) [file pcbi.1007533.s001.docx]

**Overview of the eight constraint-based models of Mtb used in this study**

**GSMN-TB1.1**

GSMN-TB 1.1 is an improved and extended version of GSMN-TB. GSMN-TB 1.1 which encompasses 759 genes, 876 intracellular reactions, 667 metabolites and additional pathways, such as the cholesterol degradation pathway and extra corrections to the original GSMN-TB model [1]. This model has the ability to predict sole substrate utilization and essential genes with high accuracy. Nonetheless, discrepancies appeared (6% of false positive and 19% of false negative genes) which required further model curation.

**iOSDD890**

Vashisht and colleagues published a curated and updated genome-scale Mtb model (iOSDD890) based on comprehensive manual re-annotation of its published genome [2]. The iOSDD890 model used iNJ661 as a basis and encompassed 890 genes, 1152 intracellular reactions and 961 metabolites. The use of this model by way of other methodologies allowed identifying metabolic persister genes and potential non-toxic drug targets. This model is part of the translational platform for drug discovery, Open Source Drug Discovery (OSDD), which is a Council of Scientific and Industrial Research (CSIR) team [3,4].

**sMtb**

Rienksma and colleagues published the first manually curated consensus genome-scale model of Mtb (sMtb). It combined the strengths of iNJ661 and GSMN-TB 1.1 and provided accurate representations of drug-phenotypes, growth rates, flux distributions, and gene essentiality predictions [5]. This model included an improved objective function and a cholesterol degradation pathway highly descriptive (36 reactions). Nevertheless, the functionality of this pathway was not validated.

**iCG760**

iCG760 is a modified version of GSMN-TB [6], in this model the biochemical pathways for production of sulfolipid-1, phthiocerol dimycocerosates, triacylglycerol, diacyltrehalose, and polyacyltrehalose were significantly improved. This model was used to validate the accuracy of E-Flux-MFC, an algorithm that allows the integration of transcriptomics and metabolomics data, specifically used for predicting changes in lipid and metabolite concentrations during hypoxia. However, this model was validated merely with old gene essentiality data [7].

**iSM810**

Ma and colleagues published iSM810 [8], is an updated version of GSMN-TB1.1 with additional information of gene-associated reactions and literature evidence of iNJ661 and HQ-Mtb that were absent in the GSMN-TB1.1. This genome-scale metabolic model integrated an extended regulatory network (composed by 104 transcription factors) and could correctly predict growth viability over 69 carbon and nitrogen sources and predict metabolic gene essentiality more accurately than the original GSMN-TB1.1 model. Although this model was validated using recent gene essentiality data [9] and carbon and nitrogen sources, no structural network metrics e.g., dead-end metabolites, and reaction gaps, were evaluated.

**iNJ661v_modified**

iNJ661v_modified is an improved version of iNJ661v [10]. Xavier and colleagues demonstrated that inclusion of universal cofactors such as NAD, NADP, COA, FAD, FMN, S-Adenosyl methionine and Pyridoxal-5-phosphate in the biomass objective function and a new biosynthetic reaction for Pyridoxal-5-phosphate in the model allowed it to increase gene essentiality predictions i.e., 7 new true predictions. Yet, this model is structurally identical to iNJ661v and highly comparable to iCG760; old gene essentiality data [7] were used to validate it.

**iEK1011**

Kavvas and colleagues built an updated and unified GEM of Mtb named iEK1011 [11]. This model combined advantages of iOSDD890, sMtb and portions of the M. tuberculosis H37Rv BioCyc Database (number of genes, reactions, and biochemical knowledge), including ergothioneine biosynthesis. The iEK1011 showed increased gene essentiality predictions in both minimal medium [9]and rich medium [12]. The use of this model allowed highlighting condition-dependent differences that may influence the efficacy of antibiotics.

**sMtb2018**

Rienksma and colleagues improved their previous Mtb model [5,13] by adding and replacing some reactions of redox metabolism, around 17 changes were made in sMtb. This model and transcriptomics data were used as platform for obtaining condition-specific biomass reactions for Mtb during relevant stages of infection, specifically *in vitro* and during infection of THP-1 cells.

**References**

1. Lofthouse EK, Wheeler PR, Beste DJV, Khatri BL, Wu H, Mendum TA, et al. Systems-based approaches to probing metabolic variation within the Mycobacterium tuberculosis complex. PLoS One. 2013;8: e75913. doi:10.1371/journal.pone.0075913

2. Brahmachari SK, Bhat AG, Kushwaha S, Bhardwaj A. Systems level mapping of metabolic complexity in Mycobacterium tuberculosis to identify high-value drug targets. J Transl Med. 2014;12: 263. doi:10.1186/s12967-014-0263-5

3. Singh S. India Takes an Open Source Approach to Drug Discovery. Cell. 2008;133: 201–203. doi:10.1016/j.cell.2008.04.003

4. Bhardwaj A, Scaria V, Raghava GPS, Lynn AM, Chandra N, Banerjee S, et al. Open source drug discovery–a new paradigm of collaborative research in tuberculosis drug development. Tuberculosis. 2011;91: 479–486.

5. Rienksma RA, Suarez-Diez M, Spina L, Schaap PJ, dos Santos VAPM. Systems-level modeling of mycobacterial metabolism for the identification of new (multi-) drug targets. Seminars in immunology. Elsevier; 2014. pp. 610–622.

6. Garay CD, Dreyfuss JM, Galagan JE. Metabolic modeling predicts metabolite changes in Mycobacterium tuberculosis. BMC Syst Biol. 2015;9: 57.

7. Sassetti CM, Boyd DH, Rubin EJ. Genes required for mycobacterial growth defined by high density mutagenesis. Mol Microbiol. 2003;48: 77–84. Available: http://www.ncbi.nlm.nih.gov/pubmed/12657046

8. Ma S, Minch KJ, Rustad TR, Hobbs S, Zhou S-L, Sherman DR, et al. Integrated modeling of gene regulatory and metabolic networks in Mycobacterium tuberculosis. PLoS Comput Biol. 2015;11: e1004543.

9. Griffin JE, Gawronski JD, DeJesus MA, Ioerger TR, Akerley BJ, Sassetti CM. High-resolution phenotypic profiling defines genes essential for mycobacterial growth and cholesterol catabolism. PLoS Pathog. 2011;7: e1002251. doi:10.1371/journal.ppat.1002251

10. Xavier JC, Patil KR, Rocha I. Integration of biomass formulations of genome-scale metabolic models with experimental data reveals universally essential cofactors in prokaryotes. Metab Eng. 2017;39: 200–208.

11. Kavvas ES, Seif Y, Yurkovich JT, Norsigian C, Poudel S, Greenwald WW, et al. Updated and standardized genome-scale reconstruction of Mycobacterium tuberculosis H37Rv, iEK1011, simulates flux states indicative of physiological conditions. BMC Syst Biol. 2018;12: 25.

12. DeJesus MA, Gerrick ER, Xu W, Park SW, Long JE, Boutte CC, et al. Comprehensive Essentiality Analysis of the Mycobacterium tuberculosis Genome via Saturating Transposon Mutagenesis . MBio. 2017;8: e02133-16. doi:10.1128/mbio.02133-16

13. Rienksma RA, Schaap PJ, Martins dos Santos VAP, Suarez-Diez M. Modeling the metabolic state of Mycobacterium tuberculosis upon infection. Front Cell Infect Microbiol. 2018;8: 264.
