# Supplementary material for: A systematic evaluation of Mycobacterium tuberculosis Genome-Scale Metabolic Networks
Source: PLoS Comput Biol. 2020 Jun 15;16(6):e1007533. doi: 10.1371/journal.pcbi.1007533 (PMC7316355; doi:10.1371/journal.pcbi.1007533)
Supplement: S2 Appendix — A document providing detailed description of the curation of TICs in sMtb2.0. (DOCX) [file pcbi.1007533.s002.docx]

**Curation of additional Thermodynamic Infeasible Cycles (TICs) in sMtb2.0**

TIC #1 was formed by two aspartate aminotransferases *aspB* and *aspC* (Rv3565 and Rv0337c, respectively) catalyzing the irreversible interconversion of oxaloacetate and glutamate into a-ketoglutarate and aspartate. We removed this TIC by lumping together both ASPB3 and ASPC into one reaction named ASPBC (Rv3565 OR Rv0337c) (S3 Fig).

TIC #2 was formed by three reactions involved in the interconversion of dTDP-6-deoxy-L-mannose and dTDP-4-dehydro-6-deoxy-L-mannose (S3 Fig), which play an essential structural role in mycobacterial cell wall integrity [1–4]. Thermodynamic computation of maximum and minimum Gibbs free energy of reaction ($\Delta_{r}G_{min}=4.3x{10}^{-6}kJ/mol, \Delta_{r}G_{max}=13.3 kJ/mol$) suggests that dTDP-4-dehydrorhamnose reductase (*rmlD*, Rv3266c) works preferentially towards the production of dTDP-6-deoxyL-mannose (S21 Table). Additionally, the lack of GPR information for the reaction DPHRAM brought us to eliminate it from the model.

Mtb succinate dehydrogenases (SDH1 and SDH2) and fumarate reductase (FRD) form the TIC #3 (S3 Fig). These enzymes are part of complex II of the respiratory chain and couples oxidative phosphorylation to tricarboxylic acid cycle (TCA) in CCM, catalyzing the oxidation of succinate to fumarate wherein two electrons are transferred to menaquinol (SDH1 or SDH2) or the reverse reaction (FRD). SDH1 genes have shown to be upregulated and essential in aerobic conditions while SDH2 and FRD genes upregulated under oxygen-limiting conditions [5]. Besides, in an oxygen-limiting environment, carbon flux from fumarate to succinate kept unchanged in an Mtb FRD deletion mutant, which suggests that SDH2 could catalyze this reaction [6]. This finding may advise the combination of FDR and SDH2 reactions without SDH1 in our sMtb2.0 model. However, if we do so, sMtb2.0 would not be able to consume fatty acids such as decanoate, laurate, or arachidic acid, due to the electron acceptor used by SDH2 (FAD), different to menaquinone, which is used by SDH1 (Fig 5). Therefore, this TIC was kept in sMtb2.0 until new findings about fumarate reductase and succinate reductase might be published.

TIC #4 involves linear dependence of the enzyme Pyrroline-5-carboxylate reductase (***proC***), which is active at the terminal (third) step in proline biosynthesis (S4 Fig.). This TIC was broken by eliminating PROC1, as we forced the use of NADPH/NADP in anabolic reactions and NADH/NAD for catabolic reactions.

TIC #5 involves two alcohol dehydrogenases, ***adhA*** and ***adhC*** (S4 Fig). This TIC was not eliminated because there is evidence of the existence of both NADP- and NAD-dependent alcohol dehydrogenases. However, our thermodynamic computation analysis suggested that ***adhC*** is irreversible in the forward direction producing ethanol ($\Delta_{r}G_{min}=-87\frac{kJ}{mol}, \Delta_{r}G_{max}=-17 kJ/mol$, S22 Table).


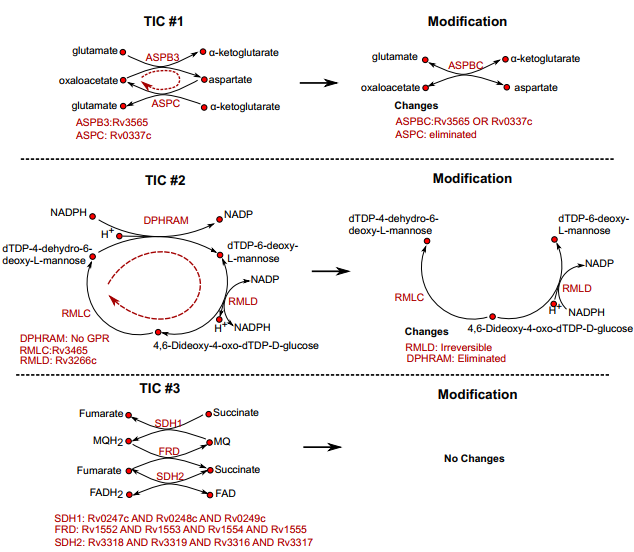


**S3 Fig.** TICS #1, 2, and 3 from sMtb2.0.

TIC #6 is formed by polyphosphate kinase (***ppk***), and polyphosphate glucokinase (***ppgK***) (S4 Fig). Breaking this TIC is challenging because of the participation of polyphosphate is poor in this Mtb network. However, we eliminated this TIC by forcing ***ppk*** to the forward direction. This change allowed the recovery of the pool of polyphosphate to be used to produce D-Glucose 6-phosphate by PPGK2.

TIC #7 is formed by two guanylate kinase (***gmk***) reactions and one nucleoside diphosphate kinase (***ndkA***). The ***gmk*** is involved in GMP of recycling whereas ***ndkA*** participates in the biosynthesis of nucleoside triphosphates other than ATP. In order to break this TIC, both ***gmk*** reactions were forced to work in the direction of GDP production as Saha and colleagues [7], and FVA fluxes, recommended (data not shown).

**
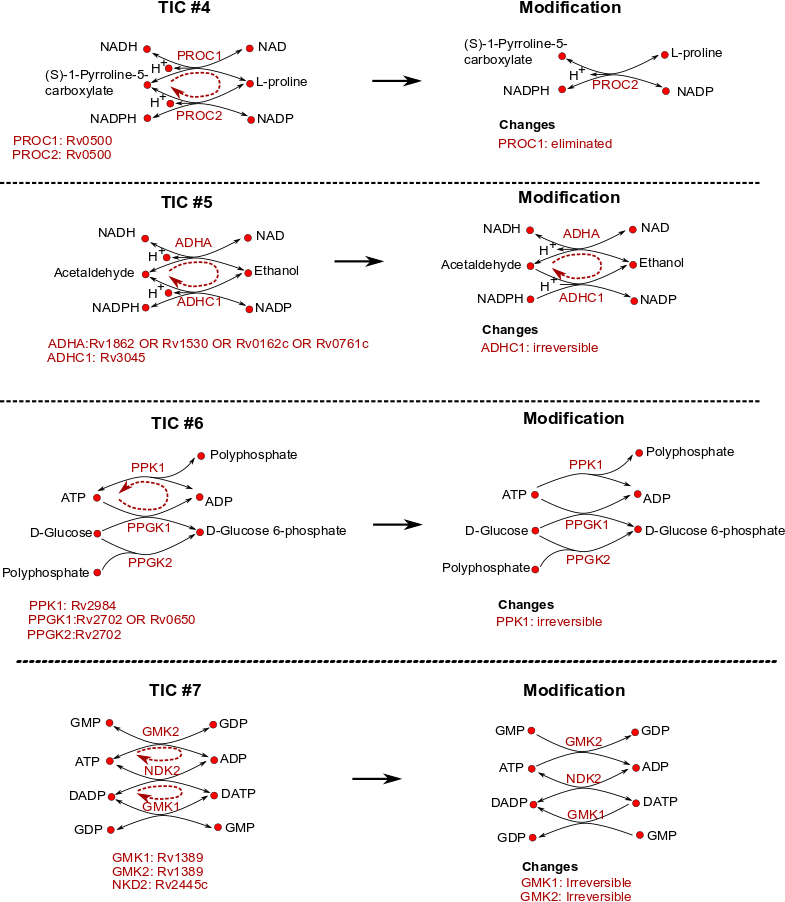
**

**S4 Fig.** TICS #4, 5, 6 and 7 from sMtb2.0.

TIC #8 involves four reactions, Cytidylate kinase (***cmk***), nucleoside diphosphate kinase (***ndkA***), phosphatidate cytidylyltransferase (***cdsA***) and CDP-diacylglycerol pyrophosphatase (***cdh***) (S5 Fig). Forcing ***cmk*** and ***ndkA*** to work in the forward direction, towards the production of ADP and CDP, and CTP, respectively, curated part of this TIC.

TIC #9 involves three reactions associated with isoprenoid backbone biosynthesis (S5 Fig). Our thermodynamic data suggest ISPH2 is irreversible in the dimethylallyl diphosphate direction. However, if we do so, we get erroneous predictions of gene essentiality for IDI and ISPH1 genes. Therefore, we decided to keep this TIC in sMtb2.0.

TIC #10 and TIC #11 involve inter-compartment reactions that transport Citrate and Succinate, and, Fumarate and Succinate, respectively (S5 Fig). We broke these TICs by eliminating the reactions TSUCCA and TSUCCB as they lack of associated cost metabolites like protons or ATP.

**
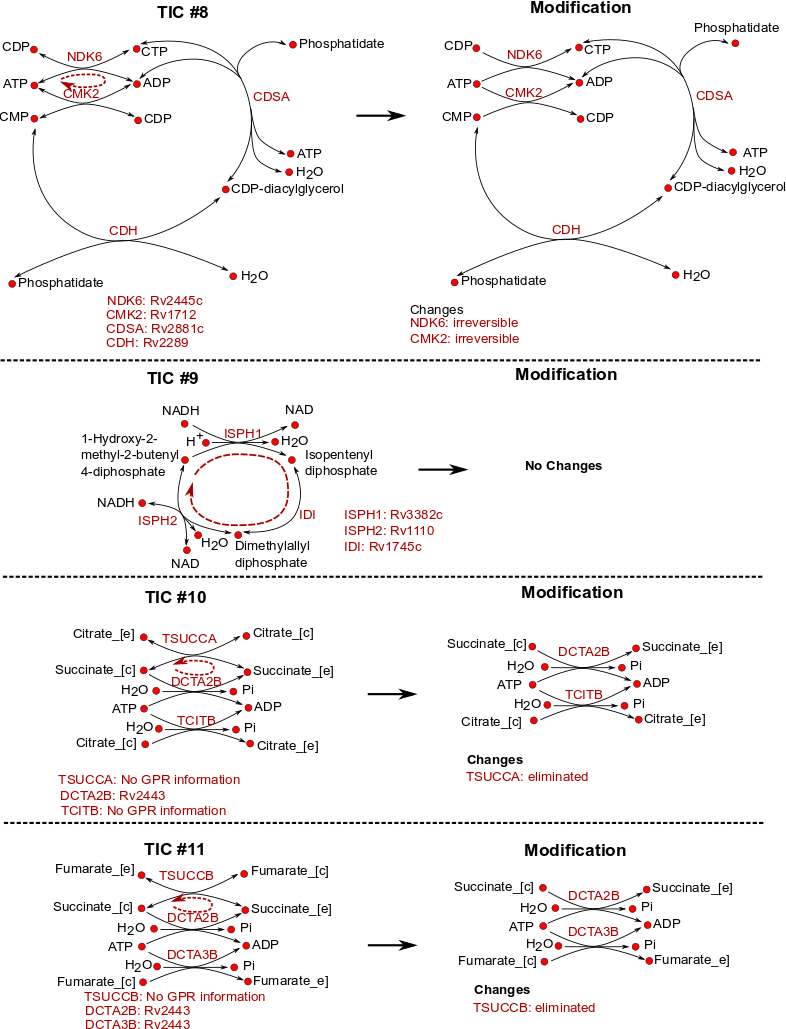
**

**S5 Fig.** TICS #8, 9, 10 and 11 from sMtb2.0.

**References**

1. Ma Y, Stern RJ, Scherman MS, Vissa VD, Yan W, Cox Jones V, et al. Drug targeting Mycobacterium tuberculosis cell wall synthesis: Genetics of dTDP-rhamnose synthetic enzymes and development of a microtiter plate-based screen for inhibitors of conversion of dTDP-glucose to dTDP-rhamnose. Antimicrob Agents Chemother. 2001;45: 1407–1416. doi:10.1128/AAC.45.5.1407-1416.2001

2. Ma Y, Pan F, McNeil M. Formation of dTDP-rhamnose is essential for growth of mycobacteria. J Bacteriol. 2002;184: 3392–3395. doi:10.1128/JB.184.12.3392-3395.2002

3. Ren JX, Qian HL, Huang YX, Zhu NY, Si SY, Xie Y. Virtual screening for the identification of novel inhibitors of Mycobacterium tuberculosis cell wall synthesis: Inhibitors targeting RmlB and RmlC. Comput Biol Med. 2015;58: 110–117. doi:10.1016/j.compbiomed.2014.12.020

4. Brown HA, Thoden JB, Tipton PA, Holden HM. The structure of glucose-1-phosphate thymidylyltransferase from Mycobacterium tuberculosis reveals the location of an essential magnesium ion in the RmlA-type enzymes. Protein Sci. 2018;27: 441–450. doi:10.1002/pro.3333

5. Berney M, Cook GM. Unique flexibility in energy metabolism allows mycobacteria to combat starvation and hypoxia. PLoS One. 2010;5: e8614. doi:10.1371/journal.pone.0008614

6. Watanabe S, Zimmermann M, Goodwin MB, Sauer U, Barry CE, Boshoff HI. Fumarate reductase activity maintains an energized membrane in anaerobic Mycobacterium tuberculosis. PLoS Pathog. 2011;7: e1002287. doi:10.1371/journal.ppat.1002287

7. Saha R, Verseput AT, Berla BM, Mueller TJ, Pakrasi HB, Maranas CD. Reconstruction and Comparison of the Metabolic Potential of Cyanobacteria Cyanothece sp. ATCC 51142 and Synechocystis sp. PCC 6803. PLoS One. 2012;7. doi:10.1371/journal.pone.0048285
