# Supplementary material for: A systematic evaluation of Mycobacterium tuberculosis Genome-Scale Metabolic Networks
Source: PLoS Comput Biol. 2020 Jun 15;16(6):e1007533. doi: 10.1371/journal.pcbi.1007533 (PMC7316355; doi:10.1371/journal.pcbi.1007533)
Supplement: S3 Appendix — A document providing detailed description of the curation of TICs in iEK1011_2.0. (DOCX) [file pcbi.1007533.s003.docx]

**Curation of additional Thermodynamic Infeasible Cycles (TICs) in iEK1011_2.0**

Four catalytic enzymes formed TIC #1 in iEK1011: glutamate dehydrogenase (GLUDxi, Rv2476c), valine transaminase (VALTA, Rv2210c), valine-pyruvate aminotransferase (VPAMTr, Rv3565) and alanine dehydrogenase (ALAD_L, Rv2780) (S6 Figure). These enzymes exchange amino acids like glutamate, a-ketoglutarate, valine and alanine, and electron carriers as NAD^+^ and NADH. With an appropriate regulation, these enzymes could be used for a fast replenishing of these amino acids or fixing ammonium concentration, based on the environmental conditions. Gibbs free energy calculations suggest that directions of these four reactions are correctly assigned in iEK1011 (S22 Table). Therefore, we do not have any strong evidence to modify or even to eliminate this TIC. Further efforts needs to be made regarding regulation of metabolic networks, as some FBA identified TICs would be modified after assigning appropriate regulatory constraints.

Two copies of the dihydrofolate reductase enzyme (DHFR, Rv2763c) formed TIC #2 (S6 Figure). Gibbs free energy calculations using NExT algorithm suggest that this enzyme is irreversible ($\Delta_{r}G_{min}=-65.26\frac{kJ}{mol}, \Delta_{r}G_{max}=-1.21\times10^{-8} kJ/mol$, S22 Table). However, as the value of $\Delta_{r}G_{max}$ is so closed to zero, we decided to hold the reversible classification. The reaction named “DHFR_copy2” contains an erroneous GPR assignation as the Rv2671 codifies for riboflavin-specific deaminase (*ribD*). Therefore, we curated this TIC by the elimination of the “DHFR_copy2” reaction.

The TIC # 3 is composed by three reactions annotated as NADPH-dependent mycothiol reductases. Although the function of this enzyme is the mycothiol reduction, one of these three reactions catalyzes glutathione reduction (GTHOr) and the other an interconversion between the oxidized and reduced forms of glutathione and mycothiol (MYCTR2) (S6 Fig). Thermodynamic information showed that the directionality of these three reactions is suitable (S22 Table). However, the literature suggests that Mtb uses mycothiol (MSH) instead of glutathione (GSH) to keep a reducing environment within the cytosol and as main anti-oxidant defense [1]. Although GSH is not the main thiol used in Mtb, in macrophages, GSH has a known role in the defense against oxygen toxicity [2]. During infection by Mtb, alveolar macrophages induce nitric oxide (NO), Reactive Nitrogen Species (RNS), Reactive Oxygen Species (ROS), and GSH production. This GSH production is apparently used to protect macrophages against the toxic effects of NO, RNS, and ROS, and to intoxicate Mtb by generation of S-nitrosoglutathione. Although Mtb should have particular mechanisms for avoiding intoxication by glutathione-derived species, these mechanisms are not clearly understood. Therefore, we decided to keep the MYCTR2 reaction (interconversion between oxidized and reduced forms of glutathione and mycothiol), as it could constitute an interesting metabolic interaction between host and pathogen whereas new evidence about detoxification of glutathione-derived species is available.


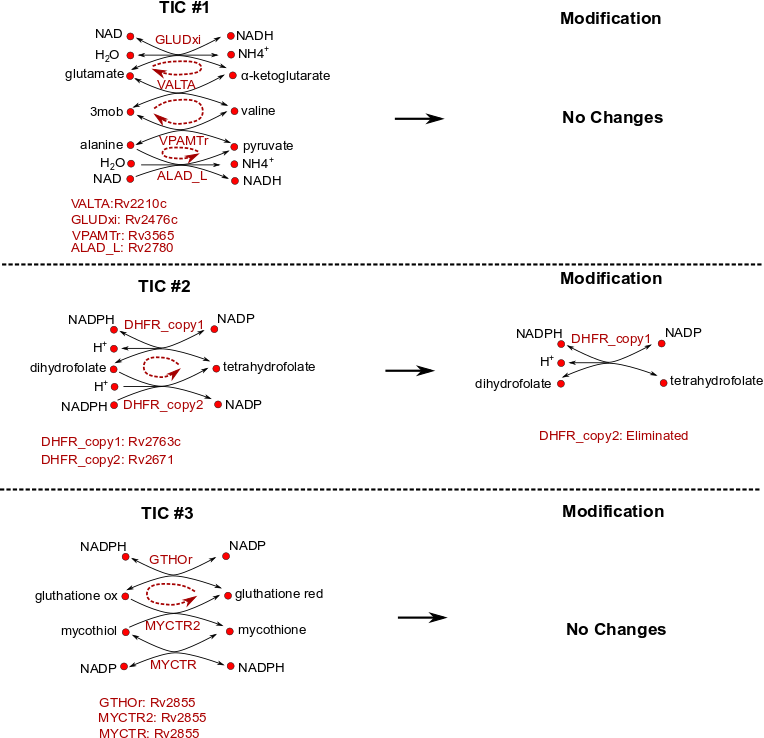


**S6 Figure.** TICs # 1, 2, and 3 from iEK1011_2.0.

Two reactions involved in potassium transport exchange formed the TIC #4; these are: the TRK system potassium uptake (Kt1, *ceoB*) and the potassium/proton antiporter protein (NAKtr, Rv3236c). As there is little experimental/functional evidence about the characteristics of these reactions, we decided to keep both reactions (S7 Fig).

Similar to those TICs found in sMtb2.0 (TIC #7 and TIC #8, S4 and S5 Figure, S2 Appendix), the iEK1011 contains a TIC involved with kinase activity (Purine and Pyrimidine biosynthesis). This TIC #5 is formed by two adenylate kinases (ADK1-ADK2, Rv0733) and a polyphosphate kinase (PPK2, Rv2984) reaction (S7 Fig). Although thermodynamics constraints show that directionality of these reactions is appropriate, Saha and Colleagues suggest that those kinase reactions should go in the direction of the less energetic currency metabolite [3], it means ADP, or AMP. Therefore, we modified the reaction ADK2 in the backward direction, towards AMP metabolite synthesis.

Finally, the TIC #6 is formed by a FAD-dependent succinate dehydrogenase (SUCD1) and FAD-dependent fumarate reductase (S7 Fig). Recent literature discusses the existence of succinate dehydrogenase and fumarate reductase menaquinone-dependents [4]. However, the absence of information denying the existence of FAD-dependent succinate dehydrogenases for Mtb and observations in BiGG database about this enzyme in genome-scale models of bacteria such as *E.coli* K-12 (iJR904) and *Pseudomonas putida* KT2440 (iJN746) would suggest keeping this TIC while new information comes out.

**
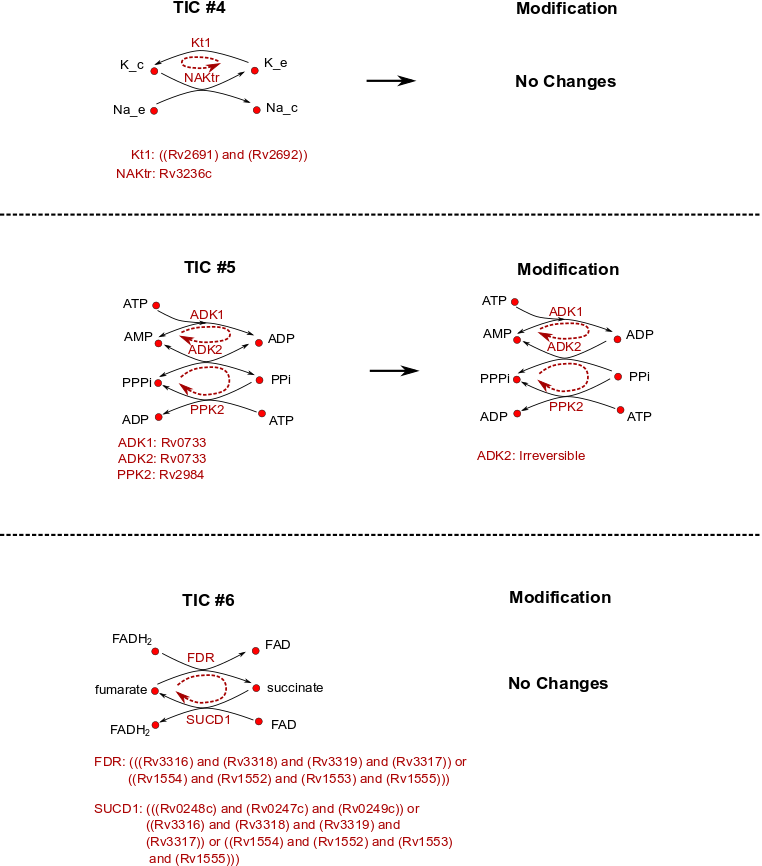
**

**S7 Fig.** TICs # 4, 5 and 6 from iEk1011_2.0

**Reference**

1. Ung KSE, Av-Gay Y. Mycothiol-dependent mycobacterial response to oxidative stress. FEBS Lett. 2006;580: 2712–2716. doi:10.1016/j.febslet.2006.04.026

2. Attarian R, Bennie C, Bach H, Av-Gay Y. Glutathione disulfide and S-nitrosoglutathione detoxification by Mycobacterium tuberculosis thioredoxin system. FEBS Lett. 2009;583: 3215–3220. doi:10.1016/j.febslet.2009.09.007

3. Saha R, Verseput AT, Berla BM, Mueller TJ, Pakrasi HB, Maranas CD. Reconstruction and Comparison of the Metabolic Potential of Cyanobacteria Cyanothece sp. ATCC 51142 and Synechocystis sp. PCC 6803. PLoS One. 2012;7. doi:10.1371/journal.pone.0048285

4. Cook GM, Hards K, Vilchèze C, Hartman T, Berney M. Energetics of Respiration and Oxidative Phosphorylation in Mycobacteria. Microbiol Spectr. 2014;2. doi:10.1128/microbiolspec.mgm2-0015-2013
