# Supplementary figures and images for: A systematic evaluation of Mycobacterium tuberculosis Genome-Scale Metabolic Networks
Source: PLoS Comput Biol. 2020 Jun 15;16(6):e1007533. doi: 10.1371/journal.pcbi.1007533 (PMC7316355; doi:10.1371/journal.pcbi.1007533)

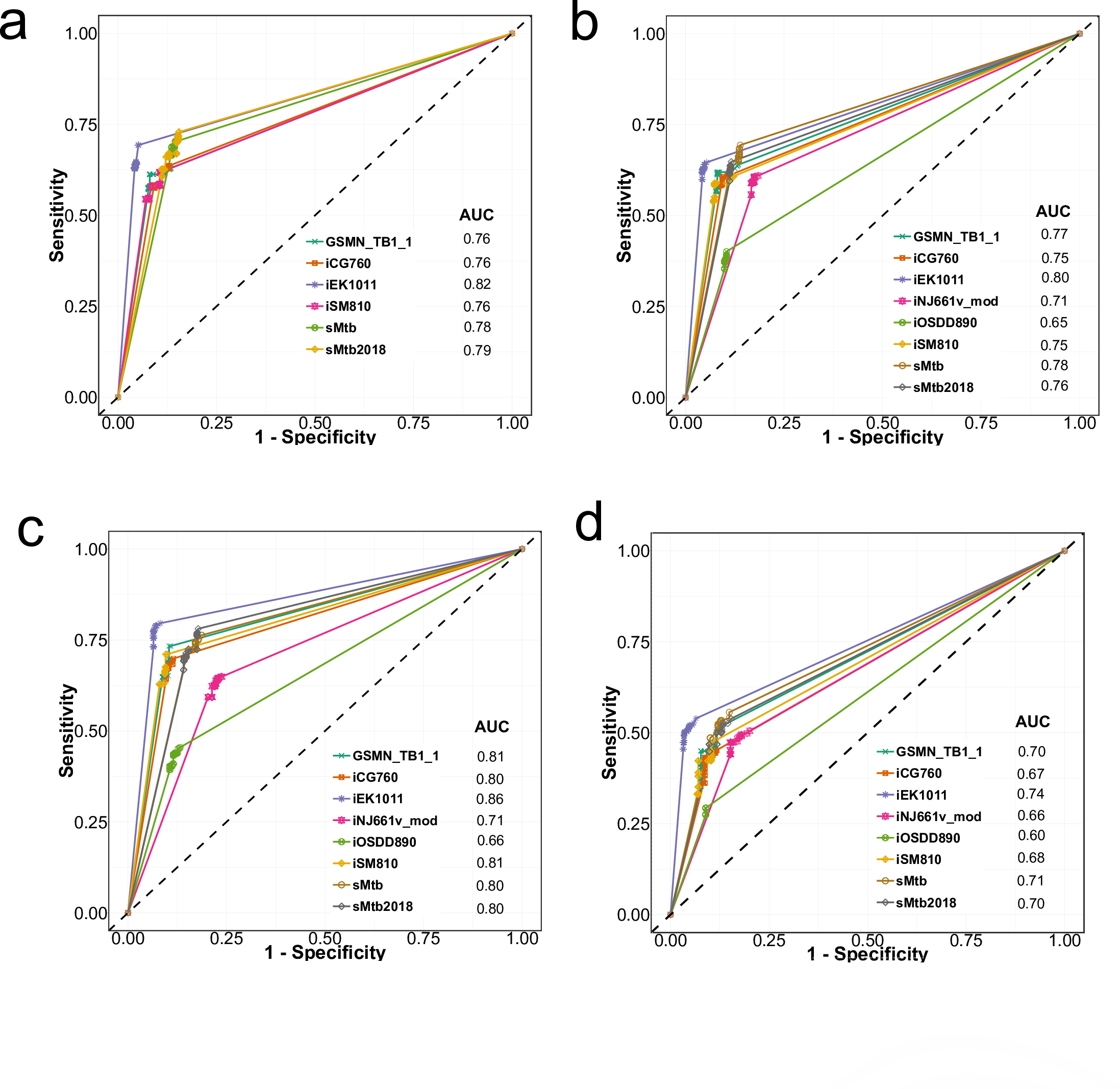

Supplement: S1 Fig — a Receiver operating characteristic curve for the gene essentiality predictions in cholesterol minimal medium, b Receiver operating characteristic curve for the gene essentiality predictions in glycerol minimal medium, c Receiver operating characteristic curve for gene essentiality predictions in 7H9 Middlebrook OADC medium, d Receiver operating characteristic curve for gene essentiality predictions in MtbYM medium. (TIF) [file pcbi.1007533.s004.tif]

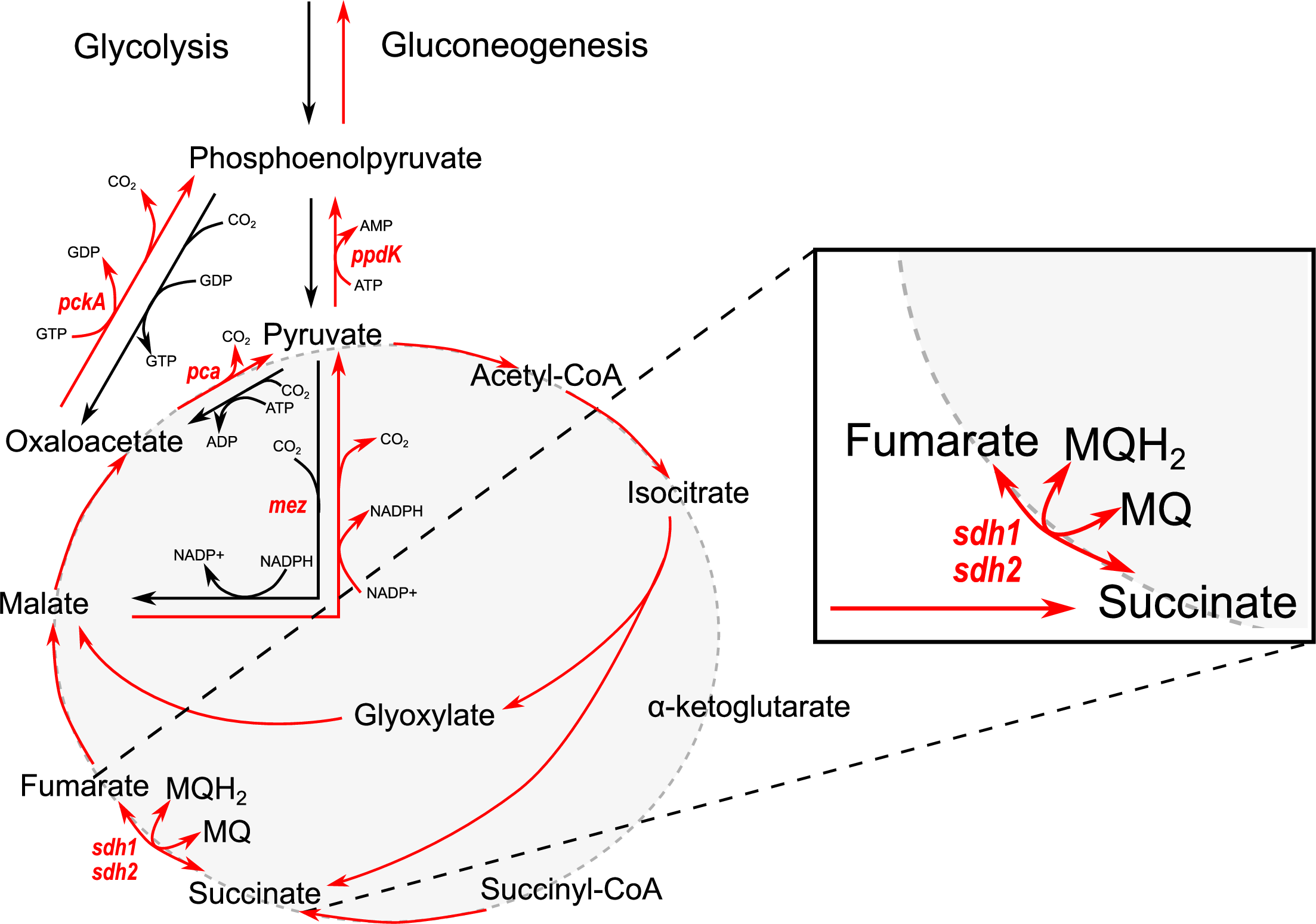

Supplement: S2 Fig — (TIF) [file pcbi.1007533.s005.tif]
