# Supplementary material for: A systematic evaluation of Mycobacterium tuberculosis Genome-Scale Metabolic Networks
Source: PLoS Comput Biol. 2020 Jun 15;16(6):e1007533. doi: 10.1371/journal.pcbi.1007533 (PMC7316355; doi:10.1371/journal.pcbi.1007533)
Supplement: S8 Table — (DOCX) [file pcbi.1007533.s013.docx]

**S8 Table.** Differences in stoichiometric coefficients of BIOMASS_2 from iEK1011 compared with default biomass objective functions of sMtb and iOSDD890

| **Metabolites** | **Name** | **SC_BIOMASS_2 (iEK1011) (mmol/gDW)** | **SC_biomass_Mtb_9_60atp (iOSDD890) (mmol/gDW)** | **SC_BiomassGrowth (sMtb) (mmol/gDW)** |
| --- | --- | --- | --- | --- |
| Ac1PIM1[c] | Acyl phosphatidylinositol mannoside (tuberculosis) | 0,001681 | 0,001681 | 0,0024 |
| Ac1PIM2[c] | Acyl phosphatidylinositol mannoside dimannose (tuberculosis) | 0,001488 | 0,001488 | 0,0014 |
| Ac1PIM3[c] | Acyl phosphatidylinositol mannoside trimannose (tuberculosis) | 0,001335 | 0,001335 |  |
| Ac1PIM4[c] | Acyl phosphatidylinositol mannoside tetramannose (tuberculosis) | 0,001211 | 0,001211 |  |
| Ac2PIM2[c] | Diacyl phosphatidylinositol mannoside dimannose (tuberculosis) | 0,001274 | 0,001274 | 0,0024 |
| PIM3[c] | Phosphatidylinositol mannoside trimannose (tuberculosis) | 0,001573 | 0,001573 |  |
| PIM4[c] | Phosphatidylinositol mannoside tetramannose (tuberculosis) | 0,001403 | 0,001403 |  |
| PIM5[c] | Phosphatidylinositol mannoside pentamannose (tuberculosis) | 0,001266 | 0,001266 |  |
| PIM6[c] | Phosphatidylinositol mannoside hexamannose (tuberculosis) | 0,001154 | 0,001154 |  |
| acgam1p[c] | NAcetylDglucosamine 1phosphate | 0,23018 | 0,23018 |  |
| adp[c] | ADP C10H12N5O10P2 | 60 | 60 | 57 |
| ala__L[c] | LAlanine | 0,40596 | 0,40596 | 0,2184 |
| amet[c] | SAdenosylLmethionine | 0,0347 |  | 0,0347 |
| arg__L[c] | LArginine | 0,12042 | 0,12042 | 0,1148 |
| asn__L[c] | LAsparagine | 0,047699 | 0,047699 | 0,0351 |
| asp__L[c] | LAspartate | 0,12031 | 0,12031 | 0,0886 |
| atp[c] | ATP C10H12N5O13P3 | 60 | 60 | 57,03 |
| c78mycolatepp[c] | C78alphamycolatePP | 0,0295 |  | 0,0295 |
| clpn160190[c] | Cardiolipin (dihexadecanoyl, dimethylstearoyl) | 0,005859 | 0,005859 |  |
| cys__L[c] | LCysteine | 0,022 | 0,022 | 0,0155 |
| damp[c] | DAMP C10H12N5O6P | 0,003492 | 0,003492 |  |
| datp[c] | DATP C10H12N5O12P3 | 0,0102 |  | 0,0102 |
| dctp[c] | DCTP C9H12N3O13P3 | 0,0194 |  | 0,0194 |
| dgtp[c] | DGTP C10H12N5O13P3 | 0,0194 |  | 0,0194 |
| dttp[c] | DTTP C10H13N2O14P3 | 0,0102 |  | 0,0102 |
| fad[c] | Flavin adenine dinucleotide oxidized | 0,0129 |  | 0,0129 |
| fdxox[c] | Oxidized ferredoxin | 0,0008 |  | 0,0008 |
| fdxrd[c] | Reduced ferredoxin | 0,0008 |  | 0,0008 |
| fe2[c] | Fe2+ mitochondria | 0,0008 |  | 0,0008 |
| fe3[c] | Iron (Fe3+) | 0,0008 |  | 0,0008 |
| fmn[c] | FMN C17H19N4O9P | 0,0222 |  | 0,0222 |
| gdpmann[c] | GDPDmannose | 0,0001 |  | 0,0001 |
| glc__D[c] | DGlucose | 0,16315 | 0,16315 | 0,4661 |
| gln__L[c] | LGlutamine | 0,05812 | 0,05812 | 0,1384 |
| glu__L[c] | LGlutamate | 0,090007 | 0,090007 | 0,1625 |
| gly[c] | Glycine | 0,33581 | 0,33581 | 0,158 |
| glyc[c] | Glycerol | 0,0184 | 0,025359 | 0,0184 |
| gmp[c] | GMP C10H12N5O8P | 0,24365 | 0,24365 |  |
| gtp[c] | GTP C10H12N5O14P3 | 0,0168 |  | 0,0168 |
| h2o[c] | H2O H2O | 60 | 60 | 57 |
| h[c] | H+ | 60 | 60 |  |
| hdca[c] | Hexadecanoate (nC16:0) | 0,23515 | 0,23515 |  |
| hdcea[c] | Hexadecenoate (nC16:1) | 0,01094 | 0,01094 |  |
| hemeA[c] | Heme A C49H55FeN4O6 | 0,0008 |  | 0,0008 |
| hemeO[c] | Heme O C49H56FeN4O5 | 0,0008 |  | 0,0008 |
| hexc[c] | Hexacosanoate n C260 C26H51O2 | 0,058352 | 0,058352 |  |
| his__L[c] | LHistidine | 0,074917 | 0,040617 | 0,0343 |
| hphthiocnylcoa[c] | HydroxyphthioceranoylCoA | 0,0064 |  | 0,0064 |
| ile__L[c] | LIsoleucine | 0,087731 | 0,087731 | 0,0642 |
| leu__L[c] | LLeucine | 0,20471 | 0,20471 | 0,1483 |
| lys__L[c] | LLysine | 0,03909 | 0,03909 | 0,0317 |
| man[c] | DMannose | 0,095065 | 0,095065 | 0,1752 |
| met__L[c] | LMethionine | 0,034889 | 0,034889 | 0,0285 |
| mql8[c] | Menaquinol 8 | 0,0118 |  | 0,0118 |
| msh[c] | Mycothiol (reduced) | 0,0131 |  | 0,0131 |
| nad[c] | Nicotinamide adenine dinucleotide | 0,0152 |  | 0,0152 |
| nadp[c] | Nicotinamide adenine dinucleotide phosphate | 0,0136 |  | 0,0136 |
| pi[c] | Phosphate | 60 | 60 | 57 |
| phe__L[c] | LPhenylalanine | 0,048102 | 0,048102 | 0,0429 |
| pro__L[c] | LProline | 0,13803 | 0,13803 | 0,0924 |
| rib__D[c] | DRibose | 0,021723 | 0,021723 | 0,0511 |
| ser__L[c] | LSerine | 0,2326 | 0,1434 | 0,0892 |
| thf[c] | 5,6,7,8Tetrahydrofolate | 0,0008 |  | 0,0008 |
| thmpp[c] | Thiamine diphosphate | 0,0008 |  | 0,0008 |
| thr__L[c] | LThreonine | 0,13571 | 0,13571 | 0,0917 |
| tre6p[c] | Alpha,alpha'Trehalose 6phosphate | 0,006491 | 0,006491 | 0,0183 |
| trp__L[c] | LTryptophan | 0,020124 | 0,020124 | 0,0218 |
| tyr__L[c] | LTyrosine | 0,031756 | 0,031756 | 0,0308 |
| utp[c] | UTP C9H11N2O15P3 | 0,0088 |  | 0,0088 |
| val__L[c] | LValine | 0,20583 | 0,20583 | 0,1373 |
|  |  |  |  |  |
|  |  |  |  |  |
|  |  |  |  |  |
|  |  |  |  |  |
|  |  |  |  |  |
|  |  |  |  |  |
|  |  |  |  |  |
|  |  |  |  |  |
|  |  |  |  |  |
|  |  |  |  |  |
|  |  |  |  |  |
|  |  |  |  |  |
|  |  |  |  |  |
|  |  |  |  |  |
|  |  |  |  |  |
|  |  |  |  |  |
|  |  |  |  |  |
|  |  |  |  |  |
|  |  |  |  |  |
|  |  |  |  |  |
|  |  |  |  |  |
|  |  |  |  |  |
|  |  |  |  |  |
|  |  |  |  |  |
|  |  |  |  |  |
|  |  |  |  |  |
|  |  |  |  |  |
|  |  |  |  |  |
|  |  |  |  |  |
|  |  |  |  |  |
|  |  |  |  |  |
|  |  |  |  |  |
|  |  |  |  |  |
|  |  |  |  |  |
|  |  |  |  |  |
|  |  |  |  |  |
