# Supplementary material for: A systematic evaluation of Mycobacterium tuberculosis Genome-Scale Metabolic Networks
Source: PLoS Comput Biol. 2020 Jun 15;16(6):e1007533. doi: 10.1371/journal.pcbi.1007533 (PMC7316355; doi:10.1371/journal.pcbi.1007533)
Supplement: S4 File — (ZIP) [file pcbi.1007533.s036.zip › S4_File/medium/2009_Roisins_Minimal_Medium.pdf]

OPEN ACCESS Freely available online

PLoS one

The Genetic Requirements for Fast and Slow Growth in Mycobacteria

Dany J. V. Beste, Mateus Espasa, Bhushan Bonde, Andrzej M. Kierzek, Graham R. Stewart, Johnjoe McFadden*

FHMS, University of Surrey, Guildford, United Kingdom

Abstract

Mycobacterium tuberculosis infects a third of the world's population. *Primary tuberculosis* involving active fast bacterial replication is often followed by asymptomatic *latent tuberculosis*, which is characterised by slow or non-replicating bacteria. Reactivation of the latent infection involving a switch back to active bacterial replication can lead to post-primary transmissible tuberculosis. Mycobacterial mechanisms involved in slow growth or switching growth rate provide rational targets for the development of new drugs against persistent mycobacterial infection. Using chemostat culture to control growth rate, we screened a transposon mutant library by Transposon site hybridization (TraSH) selection to define the genetic requirements for slow and fast growth of *Mycobacterium bovis* (BCG) and for the requirements of switching growth rate. We identified 84 genes that are exclusively required for slow growth (69 hours doubling time) and 256 genes required for switching from slow to fast growth. To validate these findings we performed experiments using individual *M. tuberculosis* and *M. bovis* BCG knock out mutants. We have demonstrated that growth rate control is a carefully orchestrated process which requires a distinct set of genes encoding several virulence determinants, gene regulators, and metabolic enzymes. The *mce1* locus appears to be a component of the switch to slow growth rate, which is consistent with the proposed role in virulence of *M. tuberculosis*. These results suggest novel perspectives for unravelling the mechanisms involved in the switch between acute and persistent TB infections and provide a means to study aspects of this important phenomenon *in vitro*.

Citation: Beste DJV, Espasa M, Bonde B, Kierzek AM, Stewart GR, et al. (2009) The Genetic Requirements for Fast and Slow Growth in Mycobacteria. PLoS ONE 4(4): e5349. doi:10.1371/journal.pone.0005349

Editor: Olivier Neyrolles, Institut de Pharmacologie et de Biologie Structurale, France

Received January 23, 2009; **Accepted** March 31, 2009; **Published** April 28, 2009

Copyright: © 2009 Beste et al. This is an open-access article distributed under the terms of the Creative Commons Attribution License, which permits unrestricted use, distribution, and reproduction in any medium, provided the original author and source are credited.

Funding: This work was supported by a grant from the Biotechnology and Biological Sciences Research Council (BBSRC), grant reference number BB/D007208/1. Mateus Espasa was supported by a grant from the Spanish Health Investigation's funds (Fondo de Investigacioes Sanitarias, FIS). The funders had no role in study design, data collection and analysis, decision to publish, or preparation of the manuscript.

Competing Interests: The authors have declared that no competing interests exist.

* E-mail: j.mcfadden@surrey.ac.uk

Introduction

With the average daily death toll from tuberculosis at 4,500 the worldwide burden of this disease is overwhelming [1]. Control measures are being severely thwarted by the worldwide spread of antibiotic resistant strains of *Mycobacterium tuberculosis*. In 2008 WHO recorded the highest rates of multi-drug (MDR) resistant strains of TB and the spread of a virtually untreatable form of the disease caused by extensively resistant strains (X-DR) [1]. Novel anti-TB drugs are urgently required which shorten the lengthy drug regimen required to treat TB and also have activity against drug resistant strains. The identification of genes which provide essential but unique functions in *M. tuberculosis* greatly facilitates drug discovery programs and also provides further information about the complex biology of this highly successful pathogen.

Slow growth rate is amongst one of the remarkable features of *M. tuberculosis*. This pathogen can only achieve a maximum growth rate equivalent to a doubling time of about 16 hours in optimal laboratory conditions whilst in the human host growth rates vary upon the site and stage of infection. Tuberculosis is characterized by two distinct phases an acute phase where the bacteria are actively growing and a persistent phase where the bacteria are in a slow growing or non-growing state [2]. This ability to persist for decades in a state refractory to immune clearance but primed for reactivation is key to the success of *M. tuberculosis* and represents an important barrier to the control of tuberculosis as presently used chemotherapies are largely inactive against non-dividing cells. The identification of pathways and genes essential to establish and maintain persistence would facilitate the development of drugs which target this phase of infection.

Although very little is known about the state of the tubercle bacillus during persistence, it is generally agreed that the organism replicates slowly or not at all during this stage of disease. To study this component of the biology of *M. tuberculosis* we utilized chemostat culture as this is the only method available to control growth rate. In addition, the use of continuous culture in a chemostat offers many advantages over batch culture enabling the experimenter to vary the growth rate whilst maintaining cells in a constant physical and chemical environment. Our previous studies into the physiology of *Mycobacterium bovis* BCG (the vaccine strain of the tubercle bacillus) demonstrated that growth rate modulated the biomass composition [3]. In addition, the transcriptomic fingerprint for slow growing BCG showed a high correlation to the profile of *M. tuberculosis* growing in a macrophage [4] and also TB patient's sputum [5].

Whilst transcriptomics has proved to be an excellent tool for studying the response of microbes to the environment it has been shown to have limited value in predicting the contribution of individual genes to the fitness of *M. tuberculosis* [6,7]. The current study aims to further probe the physiological and gene regulatory

state of slow-growing *M. tuberculosis* by mutational analysis. Transposon site hybridisation (TraSH) is a microarray based transposon tracking strategy to monitor the fitness of mutants in mixed populations under different conditions. This powerful functional genomic tool was developed using *M. tuberculosis* and has now been applied successfully to a variety of pathogens [8,9]. Sassetti and co-workers have used TraSH to identify essential genes for the growth of *M. tuberculosis* in minimal glucose medium [8] as well as infection of mice [10] and survival in a macrophage [7]. We build on this work to identify genes required for slow and fast growth on minimal glycerol media in the controlled environment of a chemostat. This data provides an invaluable resource for mycobacterial researchers and will facilitate the characterisation of genes with unknown roles and provide further functional clues for some important virulence genes.

Results and Discussion

Genes required for growth on Roisin's glycerol minimal media

In the experiments described by Sassetti *et al* (2003) transposon libraries of *M. tuberculosis* and *M. bovis* BCG were constructed and recovered on 7H10 agar and TraSH analysis was used to identify genes which were essential for the survival of both species on this media. The experiments described here used just the BCG transposon library generated by Sassetti *et al* (2003) for TraSH analysis to investigate the genetic basis of growth control in a chemostat model. Initially we determined the genes that were 'additionally essential' for growth in the defined minimal media used for chemostat cultivations: Roisin's minimal broth. The additionally essential genes discussed here are defined as genes which were identified in this study as essential for growth of *M. bovis* BCG on Roisin's minimal glycerol medium but had not been previously identified by Sassetti *et al* (2003) as essential for the growth of *M. bovis* BCG and *M. tuberculosis* on 7H10 medium.

A late log phase culture of the BCG transposon library [8] was inoculated into a 2 L bioreactor operated in batch mode. Cells were harvested after the OD_{600} reached approximately 1.0. The composition of the output mutant pools was compared by TraSH as described using randomly labeled genomic DNA as a control probe [10]. Microarray signals were analysed using a rank based statistic, Rank products (RP) [11]. RP is a non-parametric statistical method which has demonstrated robustness in microarray analysis and has been shown to have a higher sensitivity and selectivity in comparison with t-test based statistical methods [12]. This method detects genes that consistently have the largest positive or negative log-ratio.

Roisin's media is a chemically defined glycerol-limited media that contains only one source of carbon plus Tween 80 as a dispersal agent, ammonia as a nitrogen source and lacks citrate, biotin or pyridoxine [3]. In contrast 7H10 is composed of several potential carbon sources including glutamic acid, glycerol, glucose, oleic acid and bovine serum albumin and also contains the co-factors biotin and pyridoxine hydrochloride and the buffer sodium citrate facilitating citrate mediated iron transport. In the absence of citrate mycobacteria grown on Roisin's would be reliant on the siderophore mycobactin for iron transport. We therefore expected to find that the set of 'additionally essential' genes required to grow on Roisin's media to be principally those involved in glycerol uptake and metabolism, ammonia uptake and genes required for *de novo* biosynthesis of biotin, pyridoxine and mycobactin.

A total of 241 genes (Dataset S1) that were not essential for survival in 7H10 media [8] were identified here as uniquely required for growth in Roisin's minimal medium. Several of these, including *MmpL3*, *ideR*, *embA*, have previously been shown to be essential for the growth of *M. tuberculosis* on media containing both glucose and glycerol [13,14] so it is unclear why they were identified as non-essential in the original screen performed by Sassetti and colleagues (2003).

Under laboratory conditions glycerol is the favored carbon source of *M. tuberculosis* and the vaccine strain *M. bovis* BCG. *Mycobacterium bovis*, however is unable to use glycerol as a sole carbon source as a result of a single nucleotide polymorphism in the gene *pykA* [15]. During the creation of *M. bovis* BCG the serial passaging of *M. bovis* on glycerinated medium selected for the correction of this mutation [15]. The route for glycerol utilization is generally assumed to proceed *via* glycerol kinase followed by dehydrogenation [15]. However, many bacteria utilize an alternative pathway whereby glycerol is first oxidized by glycerol dehydrogenase before being phosphorylated [16]. Glycerol dehydrogenase activity has been detected in *M. tuberculosis* [17], but no gene encoding this activity has been annotated in the genome and several genes encoding putative alcohol dehyrogenases are also present. The results presented here show that *glpK* was essential for growth on Roisin's minimal glycerol media ($p = 1.48 \times 10^{-2}$). To clarify the situation, and also to independently validate the TraSH data, an individual gene knock-out of *glp*K was constructed in both *M. bovis* BCG and *M. tuberculosis*. The resulting mutants displayed a dysgonic growth phenotype when grown on 7H11 media and failed to grow on Roisin's agar (Figure 1) confirming the TraSH result and indicating that the glycerol kinase pathway is indeed essential for glycerol metabolism.

The experimental data indicated that, amongst biotin biosynthetic genes, only *bioD* was essential for growth in the biotin free Roisin's media. However, *bioA* with a pfp value of 0.114 was just below the cut-off value for essentiality. Surprisingly none of the genes involved mycobactin synthesis were essential for growth in Roisin's minimal medium. *M. tuberculosis* is peculiar in its ability to produce both cell-associated (mycobactin) and secreted (carboxymycobactin) siderophores to capture iron. The cross feeding of the *mbt* mutants by carboxymycobactin released from neighboring cells may enable *mbt* mutants to overcome their siderphore deficiency. However *mbtB*, which, encodes the enzyme required for the initial steps in mycobactin T synthesis, had a pfp value of 0.21 and therefore may have a role in the optimal growth of mycobacteria in this media. Deletion of the *mbtB* gene has been shown to disrupt the biosynthesis of both mycobactin and carboxymycobactin [18]. Cross feeding could also enable pyridoxine synthesis mutants to overcome the inability to synthesise this co-factor.

Comparison of glycerol essentiality results with flux balance analysis (FBA) model predictions of a genome-scale metabolic model

Genome-scale metabolic models provide a valuable framework to interpret essentiality screens as they allow predictions of gene essentiality using current knowledge of the metabolic network whilst also providing an insight into incomplete or incorrect metabolic knowledge. The glycerol TraSH results were compared with gene essentiality predictions using a genome-scale metabolic network of *M. tuberculosis* (GSMN-TB) [19]. The gene essentiality scan was performed using the minimal biomass e as described previously [19]. In addition the carbon source was changed to glycerol and the biotin and citrate gates were closed. Overall there was a good correlation between the GSMN-TB model and the observed gene essentialities as 76.66% of the predictions were identical to the experimental results (additionally essential genes only). This analysis identified some interesting inconsistencies (false

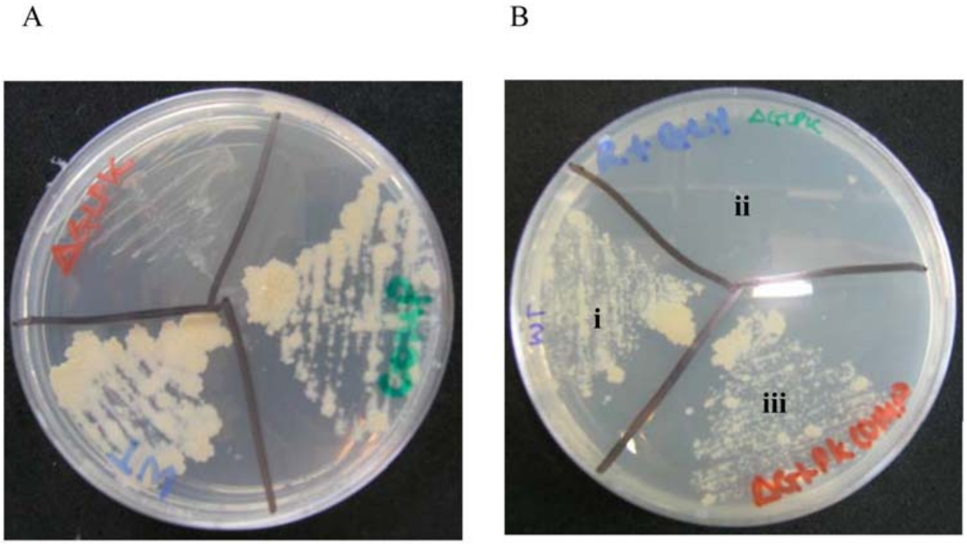

Figure 1. Growth of wild type and *glpK* mutant strains. Colonial morphology of 3 week grown (i) wild type *M. bovis* BCG (ii) a *glpK* deletion mutant and (iii) *glp*K mutant carrying a copy of the *glpK* gene in the *attB* site on the chromosome on (A) 7H11 media and (B) Roisin's glycerol minimal media. The *glp*K mutant exhibited dysgonic growth on 7H11 medium and was unable to grow on Roisin's minimal media. The wild type phenotype was restored in the complemented strain.
doi:10.1371/journal.pone.0005349.g001

positives and false negatives) which reveal important information about the metabolism of tuberculosis and this information can also be used to make informed alterations to the GSMN. The set of false negatives (genes predicted as dispensable by the model but shown to be essential experimentally) included genes which had potential homologues with annotations of only moderate confidence and maybe unable to replace the activity of their deleted isoenzyme. For example, *fabD* which is a major component of *M. tuberculosis* fatty acid synthase II has a possible homologue *fabD2* which is present in the GSMN. The demonstration that *fabD* is essential suggests that *fabD2* is unable to functionally complement the activities of *fabD*. Several genes were also predicted as functionally redundant due to the presence in the model of alternative pathways that can substitute for genes predicted to be essential. For instance, the model predicted that the gene *glpK* is non-essential due to the presence of the alternative glycerol pathway described above. This alternative glycerol utilization pathway has now been deleted from the GSMN-TB model.

Amongst the false negative model predictions was the malate dehydrogenase gene *mdh* (Rv1240), which was identified previously as non-essential in the original TraSH screen [8] but was identified as essential in the TraSH analysis presented here. The non-essentiality in the model was due to the predicted activity of "malic enzyme" (malate dehydrogenase decarboxylating, *mez*) that catalyses the oxidative decarboxylation of L-malate to pyruvate and which, together with pyruvate carboxylase, can potentially convert malate to oxaloacetate and thereby complement *in-silico mdh* mutants. To confirm this result we attempted to construct *mdh* KO mutants in both *M. tuberculosis* and *M. bovis* BCG. Despite numerous attempts we were unable to delete the *mdh* gene even when the transformants were recovered on 7H11 media which contains glucose in addition to glycerol. When a second copy of the wild type *mdh* gene was integrated into the *att*B site in the chromosome, we could easily isolate mutant strains with deletions in the original *mdh* gene demonstrating that this gene was essential for growth on both glucose and glycerol containing medium. The result indicates that either malic enzyme and/or pyruvate carboxylase are not active in Roisin's medium (at least in the direction pyruvate to acetate). To date, activity of these enzymes has not been demonstrated in *M. tuberculosis*.

Rv1099c, a fructose 1, 6 bisphosphatase (*glpX*) was predicted as essential for gluconeogenesis by the GSMN but this was not confirmed experimentally by the TraSH analysis. Rv1099c has been shown to have GlpX activity and can complement an *E. coli* mutant lacking fructose 1, 6 bisphosphatase [20]. In addition, Rv1099c was identified as essential for *in vivo* growth in a mouse model of TB by TraSH analysis [10], which is consistent with the requirement for gluconeogenesis *in vivo*. It is therefore puzzling why it is not required for growth on glycerol *in vitro*. The Rv2131c gene has been shown to possess fructose-1, 6-bisphosphatase activity in addition to inositol monophosphatase activity and could therefore potentially substitute for *glpX* [21].

Genetic requirements for growth in a carbon-limited chemostat

In order to identify genes that are important for both the control and establishment of fast and slow growth rates competitive chemostat cultivations were performed. The experiments were carried out at two different growth rates ($D = 0.03\ h^{-1}$ and $D = 0.01\ h^{-1}$) equivalent to doubling times (t_d) of 69 h and 23 h respectively. The physiological and transcriptomic profile of BCG cells at these growth rates have been described previously [3,4]. Subsequently, continuous cultures at a dilution rate of $0.03\ h^{-1}$ were switched to a dilution rate of $0.01\ h^{-1}$ and *vice versa* (Figure 2). This strategy was used to identify genes important for the change from one growth rate to another. The cultures were sampled in the batch phase just prior to the start of continuous culture and after ~ 11 generations of chemostat culture. DNA was extracted from the surviving bacterial cells and mutant gene identity and abundance assessed by TraSH. This analysis identified mutants which were negatively selected for during either slow (F) (td = 69 h) (Dataset S2, Figure 3) or fast (S) (td = 23 h) (Dataset S3, Figure 3) rates and also mutants attenuated in the switch from fast to slow (F–S) (Dataset S4) or slow to fast (S–F) (Dataset S5).

There was very little overlap between the mutants attenuated at fast growth rate and slow growth rate (Figure 3) but five genes

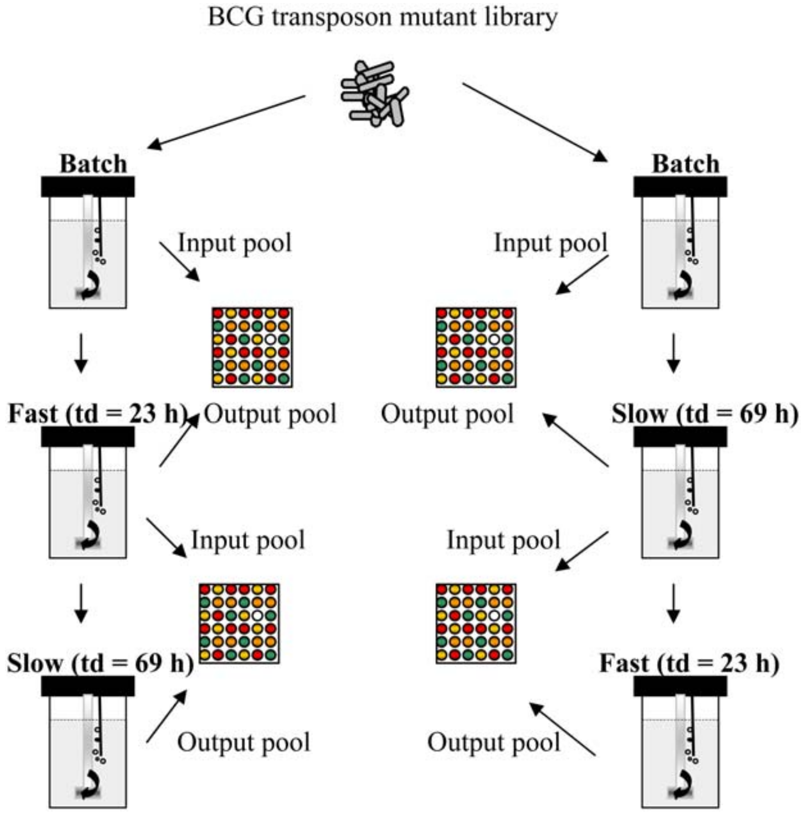

Figure 2. TraSH screen for mutants with reduced fitness at different growth rates in a carbon limited chemostat. A transposon mutant library of *Mycobacterium bovis* BCG was inoculated into the chemostat and after an initial phase in batch culture was grown at a doubling time of 23 h ($D = 0.03\ h^{-1}$) and samples removed for analysis by transposon site hybridisation (TraSH). The transposon mutant library was similarly inoculated into the chemostat at a growth rate corresponding to a doubling time of 69 h ($D = 0.01\ h^{-1}$) and samples again removed for TraSH analysis. Finally, cultures established at the fast growth rate were switched to the slow growth rate, and *visa versa*, to identify mutants unable to switch between the different growth rates.

doi:10.1371/journal.pone.0005349.g002

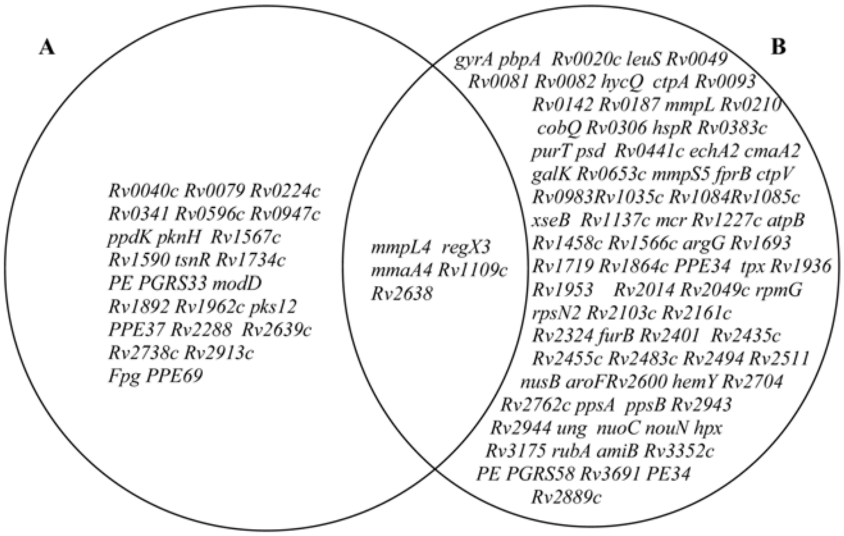

Figure 3. Mutants with reduced fitness at (A) fast ($t_d = 23$ h) and (B) slow ($t_d = 69$ h) growth rate in a carbon limited chemostat. Venn diagram shows the overlapping genes.

doi:10.1371/journal.pone.0005349.g003

were required for optimal growth in the chemostat irrespective of the growth rate (Figure 3). These included *reg*X3, *mmp*L4 and *mma*A4 which have all been shown previously to have an important role in the virulence of *M. tuberculosis* in a mouse model but without any observed alteration in their *in vitro* growth rate. The finding that they are also required for optimal growth in the chemostat suggests that they play a role in maintaining growth in carbon-limiting conditions. A very subtle growth defect during the stationary phase of growth was reported for the *reg*X3 deletion mutant of *M. tuberculosis* [22] when grown in Dubos medium in which carbon would also have been limiting growth during stationary phase. RegX3 appears to play a role in the regulation of phosphate acquisition in *Mycobacterium smegmatis* [23] but our result provides evidence of a broader regulatory role for this gene in *M. tuberculosis*. Sassetti *et al*, 2003 listed the transport protein MmpL4 as essential for *in vitro* growth; however it was demonstrated by Domenech *et al* (2005) that the individual deletion strain had no *in vitro* phenotype but had both impaired growth kinetics during the acute phase of infection and also impaired lethality in a mouse model [14]. Although several members of the MmpL group are involved in the transport of lipids the substrate of MmpL4 is unknown. It has been postulated that this protein is involved in transporting host dipeptides [14], however the demonstration here that MmpL4 is essential for growth in the minimal Roisin's media containing neither lipid nor peptides indicates that it is likely to have another function.

Genes required for slow growth rate

TraSH analysis of slowly growing BCG identified 89 genes (Dataset S2; Figure 3) important for the establishment and maintenance of this growth rate. As has been reported previously there was a very poor correlation between the transcriptome of slowly growing BCG and the TraSH essentiality data [6,7]. Only 9 genes (*cobQ1*, *purT*, *fprB*, *ctpV*, Rv1227c, Rv2014, *rpmG*, Rv2943, Rv3691) were required for slow growth rate that had also been identified as transcriptionally upregulated in this condition [19].

Essential genes were divided into functional categories using the gene ontology developed as part of the genome sequencing project (http://www.sanger.ac.uk/Projects/M_tuberculosis/Gene_list/) and a chi square test used to calculate functional groups with a significant fraction of genes identified as being required for slow growth. Nine functional categories were highlighted as being significantly involved in slow growth: *electron transport; ATP-proton motive force; cobalamin biosynthesis; modification of fatty and mycolic acids; degradation of RNA; conserved membrane proteins; detoxification; virulence and other IS elements.*

Several genes that appeared to be required for slow growth are of particular note (Table 1). Genes encoding the pin domain proteins (Rv2103c and Rv2494) were identified with mutants that had reduced fitness at slow growth rate. These proteins are putative toxin genes and have been proposed to play a role in promoting slow growth during stressful environments [24]. Interestingly, mutants in two anti-toxin genes (Rv0596c and Rv1962) were identified as having reduced fitness at the fast growth rate. Several of the genes apparently involved in slow growth have previously been associated with virulence, such as (*pps*A and *pps*B) which are essential for the synthesis of pthioceral dimycocerosates (DIM). DIM mutants have high cell wall permeability and are severely attenuated in mouse models of TB [25,26]. Several known regulators were also identified as being involved in slow growth including the heat shock protein repressor gene *hsp*R. Strains of *M. tuberculosis* lacking *hsp*R over-express heat shock proteins and have defects specifically during the chronic or persistent phase of TB infection, a feature which was proposed to be due to enhanced immune recognition [27]. To verify the involvement of this regulator in maintenance of slow growth, competitive chemostat experiments were performed using *M. bovis* BCG strains (wild type and Δ*hsp*R). The strains grew identically during the batch phase (results not shown) but had a significantly reduced fitness during slow growth rate, independently confirming the TraSH data (Figure 4). However, when the experiment was repeated using wild type *M. tuberculosis* H37Rv and a *M. tuberculosis hsp*R KO mutant [27] the mutation conferred a growth defect relative to the wild type strain during the batch phase of growth whereas there was no additional reduction of fitness observed during chemostat culture at slow growth rate (Figure 4). The result appears to indicate a difference in growth control between *M. tuberculosis* and *M. bovis* BCG.

Genes required for fast growth rate

Only 29 genes were attenuated for survival at the fast growth rate ($D = 0.03\ h^{-1}$). As the maximum growth rate (μ_{max}) of *M. bovis* BCG in Roisin's medium in continuous culture is 0.033 {Beste, 2005 1564/id) the fast growth rate is very close to μ_{max} and therefore fewer additionally essential genes would be expected. With this relatively small number of genes it was not possible to find any significant functional associations.

Genetic requirements for the shift from slow to fast growth rate

A total of 256 genes were identified as important in the switch from slow growth rate to fast. Significant functional categories

Table 1. Selected *Mycobacterium bovis* BCG mutants that incurred a high fitness cost during continuous culture in a carbon-limited chemostat at different growth rates.

Input Pool	Output pool	Gene Name and Function
Batch	Fast growth rate ($t_d = 23$ h)	**Rv0596c** (antitoxin), ***pknH*** (serine-threonine protein kinase), **Rv1962** (antitoxin)
Batch	Slow growth rate ($t_d = 69$ h)	***hspR*** (heat shock regulator), **Rv2103c** (toxin), **Rv2494** (toxin), ***ppsA*** and ***ppsB*** (DIM biosynthesis and important in virulence), ***nouC*** and ***NuoN*** (aerobic/anaerobic respiration), ***cmaA2*** (cyclopropane-mycolic acid synthase)
Batch to Fast growth rate	Slow growth rate	**Rv0485** (transcriptional regulator), ***MprA*** (mycobacterial persistence regulator), ***NuoE*** (aerobic/anaerobic respiration), ***cmaA2*** (cyclopropane-mycolic acid synthase, mutant hypervirulent in mice) ***NuoE*** (aerobic/anaerobic respiration)
Batch to Slow growth rate	Fast growth rate	**Rv0172** and ***lprK*** (part of *mce1* operon), ***dosT*** (histidine kinase sensor for Dos regulon) and ***sigF***, ***sigJ***, (alternative sigma factors involved in virulence and persistence), ***whiB1*** (transcriptional regulatory protein)

doi:10.1371/journal.pone.0005349.t001

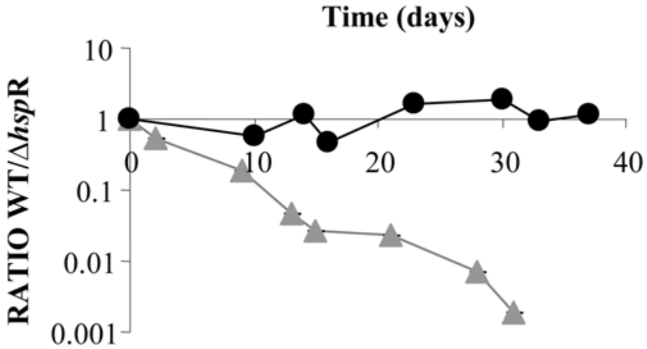

Figure 4. Competitive chemostat experiments: Wild type *Vs* *Δhspr*. Average CFU ratios of (i) wild type H37Rv+Δ*hsp*R (closed circles) and wild type BCG+Δ*hsp*R (closed triangles) during continuous culture at a dilution rate of 0.01 h^{-1} (t_d = 69 h) were plotted as a function of time. Data were normalized so that the ratio at the start of chemostat culture equaled 1. Only the BCG Δ*hspR* strain was attenuated for slow growth rate in the chemostat.
doi:10.1371/journal.pone.0005349.g004

included *energy metabolism, miscellaneous oxireductases and oxygenases, PPE family* and also the *unknown function* class indicating that a large number of uncharacterized gene products are required for the shift from slow to fast growth rate. Amongst these genes are several regulators including the stress response sigma factors *sigF* and *sigJ* and the transcriptional regulators *whiB1* and *whiB6.* Although previous studies have failed to find an *in vitro* growth effect for either *sigF* [28,28] or *sigJ* [29] mutants our results indicate that the genes are involved in the switch from slow to fast growth rate. *Sig*F mutants have reduced lethality in both mouse [28] and guinea pig models [30] of TB and also have altered cell membrane properties [31,32] whereas *sig*J is dispensable for *in vivo* growth but appears to be involved in protection against hydrogen peroxide [29]. The resuscitation promoting factor gene *rpf*E (Rv2450c) was also identified as being essential for the switch from slow to fast growth rate. Resuscitation growth factors have been implicated in the reactivation of dormant mycobacteria [33] and may also play a role in virulence [34]. *Rpf*E is one of five resuscitation factors identified in *M. tuberculosis* and although initial studies suggested functional redundancy more recent research suggests that these genes may have some degree of functional specialization [34], a notion supported by our data. RpfE has been shown to interact with an endopeptidase and probably has a role in peptidoglycan hydrolysis during cell division [35], a process that is likely to be involved in the switch from slow to fast growth rate.

Fast to slow growth rate

Only 19 genes had reduced fitness during the shift from fast to slow growth rate. Of note amongst this list was *mpr*A, a two component regulator which has been shown to have a role in the entry and maintenance of a persistent infection in mice [36]. Absence of *mpr*A also increases growth in resting macrophages [36] and increases the expression of stress related genes [37].

Positive fitness effects

Some mutations led to enhanced growth and are therefore positively selected in the competitive growth experiment. These mutants were identified using rank products, ranking on the largest positive log ratio. This generated lists of genes whose loss led to a significant selective advantage over the wild type strain in each condition (Dataset S6, S7, S8, S9). The most striking feature of this data was that for three of the four conditions examined (slow, fast and the switch from fast to slow but not the switch from slow to fast growth) almost all the top-ranked genes belong to the *mce1* operon. This was not due to simple overgrowth of a single mutant since independent mutants representing inactivation of at least 11 of the 13 genes in the *mce1* operon were over-represented in these conditions. In addition mutations in the putative ATPase, Rv0655 which has been functionally linked to the *mce1* operon [38] and also Rv0199 and Rv0200 which are closely related to genes present in the *mce1* operon [39] also had a positive fitness affect. It appears therefore that inactivation of any of the genes in the *mce1* operon and their associated genes give rise to mutants with the same competitive growth advantage.

This result seemed initially to be contradictory. Why would *mce1* operon mutants be advantageous at slow, during the switch to from fast to slow growth rate and at fast growth rate but not for the switch from slow to fast growth rate? The common factor in these three conditions is a switch from a fast to a slower growth rate. This is still compatible with the finding that the mutants also had a growth advantage at fast growth rate in the chemostat since this experiment involved a shift from growing at maximal growth rate in batch to the fast growth rate in the chemostat (which is nevertheless slower than the rate in batch). To validate this result a *M. tuberculosis mce1* operon mutant was constructed (see methods) and competitive chemostat experiments performed at the slow growth rate after an initial period in batch. The results of two independent experiments showed that although there was no difference in fitness between the wild type and mutant during batch culture (results not shown) the mutant had dramatic competitive advantage at slow growth rate in the chemostat as evidenced by the outgrowth of the *mce1* mutant from a starting mutant:WT ratio of 1 to a ratio of 100 after 35 days of continuous culture confirming the TraSH data (Figure 5). After 35 days a "quasi steady state" was obtained between the wildtype and mutant, a phenomenon which has been described previously in chemostat cultures [40]. The reasons for the maintenance of competing strains in the chemostat remains controversial and is subject to continuing mathematical investigation [41,42]. When the dilution rate was then switched to a fast growth rate the selective advantage was lost, the wild type recovered and both strains returned to approximately equivalent amounts (Figure 5). The ability of the wild type to recover at fast growth rate in this competition was intriguing and unexpected. However, the system becomes dynamic after the shift in the dilution rate and so the growth rate of both strains will depend on factors not apparent in steady state cultures. These observations may provide a clue to the function of the *mce1* operon but require further focused investigations.

The *mce1* operon has been the centre of much research and debate. Interest in this operon dates back to 1993 when Arruda *et al* demonstrated that *mce1A* was able to transfer to *Escherichia coli* the ability to invade eukaryotic cells [43]. The genome sequencing project revealed that the *mce1A* gene is part of an operon of 13 genes and that this operon is duplicated four times (*mce2, mce3, mce4*) in the genome of *M. tuberculosis* [44]. The role of the genes in *M. tuberculosis* remains highly controversial although there is an increasing consensus that each operon (which resembles a multi-subunit ABC transport system) encodes a transporter system. The *mce4* locus has recently been shown to be involved in the transport of cholesterol [38,45]. However, the role of *mce1* remains mysterious. Riley and colleagues have reported a hypervirulent phenotype for *mce1* gene knock-outs in a murine model of TB [46,47]; whereas a transposon *mce1* mutant used by Sassetti and Rubin (2003) and also Joshi *et al* (2006) [38] had a growth defect in early infection in competition experiments. An *in vitro* phenotype

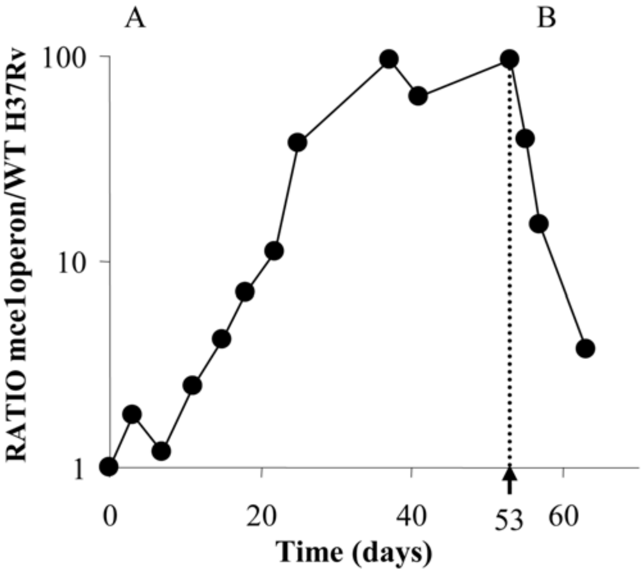

Figure 5. Competitive chemostat experiments: Wild type *M. tuberculosis* Vs Δ*mce1* operon. (A) Average CFU ratios of wild type H37Rv and Δ*mce1* operon during continuous culture at a dilution rate of 0.01 h^{-1} (t_d = 69 h) were plotted as a function of time. Data were normalized so that the ratio at the start of chemostat culture equaled 1. The *mce1* operon mutant had a competitive advantage over the wild type strain at slow growth rate. The graph is representative of two independent experiments. (B) At day 53 the dilution rate was altered to 0.03 h^{-1} (t_d = 23 h). At fast growth the Δ*mce1* did not have a competitive advantage over the wild type strain and the ratio returned to 2 after only 11 days at a dilution rate of 0.03 h^{-1} (t_d = 23 h).
doi:10.1371/journal.pone.0005349.g005

for *mce1* mutants has not previously been reported but our results show that *mce1* mutants have a competitive advantage over wild type strains during the switch to slow growth rate in a carbon limited chemostat.

Our finding that *mce1* mutants are overrepresented in slow growth rate cultures is both intriguing and puzzling. The finding has some similarities to 'evolutionary cheating' in *E. coli* whereby mutants that fail to enter, or exit early from, the non-dividing stationary phase state are overrepresented in stationary phase cultures [48]. We posit a similar scenario for *M. tuberculosis* whereby (for unknown reasons) *mce1* mutants are unable to enter, or exit early from, the slow growth rate state and are thereby overrepresented in slow growth rate cultures. This hypothesis is consistent with data showing that *mce1* expression is turned off initially during murine infection but is then switched back on in a later stage of infection [49]. If this hypothesis is correct then the underlying mechanism may be involved in the observed hypervirulent phenotype of the *mce1* knock out strain *in vivo* [46] and could have implications for understanding the switch from acute to persistent infections in the host.

Conclusion

The data presented here demonstrate that maintaining growth at slow and fast growth rate and switching between these states is a carefully controlled process in mycobacteria involving a unique set of genes and not simply acceleration or deceleration of the same cellular processes. Several of these genes are transcriptional regulators (such as *hspR*) and many have previously been implicated as involved in virulence including persistence. The results indicate that growth control in *M. tuberculosis* is a complex process and that the phenomenon of growth control, as measured in the chemostat, may have relevance to virulence (including persistence) in the host. Many of the genes that we identified as involved in growth control in the chemostat have not previously been associated with any phenotype *in vitro* and therefore the results potentially also provide a fruitful experimental route towards unraveling the role of *M. tuberculosis* genes whose function is currently unknown. Perhaps the most surprising finding of this study was the demonstration that the *mce1* operon is involved in growth control of *M. tuberculosis*. This result is very interesting but begs the question: how does *mce1* influence growth? Whilst our experiments do not identify what this role is they do provide a means to study this phenomenon *in vitro*.

Materials and Methods

Chemostat cultivations

A library of transposon mutants constructed in *M. bovis* BCG Pasteur was kindly provided by Eric J. Rubin [8]. An aliquot of the transposon insertion library was cultured in Roisin's minimal medium, without the addition of biotin [3] until an OD_{600} of 1.0. This pre-culture was transferred into a 2 L bioreactor as previously described [3]. After inoculation the culture was grown as a closed system until the OD_{600} reached approximately 1.0. Continuous culturing was then started at a known dilution rate of 0.03 h^{-1} (equivalent to a doubling time [t_d] of 24 h) or 0.01 h^{-1} (t_d = 69 h). Six to eight volume changes were allowed for selection of mutants with growth defects or enhanced survival at each dilution rate. Culture samples were harvested from the chemostat in the batch phase and during continuous culture and used to isolate genomic DNA. Cultures established at the fast growth rate were switched to the slow growth rate, and *vice versa* in order to identify mutants unable to switch between different growth rates (Figure 1). Genomic DNA was extracted from independent triplicate or quadriplate chemostat cultures using standard methods. TRASH probes were generated using the protocol described by Sassetti *et al* (2001).

For the competitive chemostat cultivations of individual mutant versus wild type equivalent amounts of antibiotic tagged mutant and wild type strains were inoculated into bioreactors and grown at a dilution rate of 0.01 h^{-1} or 0.03 h^{-1}. Samples were removed from the chemostat once every generation during continuous culture. The numbers of wild type and mutant bacteria were determined by plating serially diluted samples onto 7H11 agar (for total bacterial counts) and 7H11 agar containing hygromycin at 50 μg ml^{-1} (to enumerate the cfu of the mutant).

Microarrays and hybridizations

Fluorescently labeled cDNA were produced by reverse transcription of the TRASH probes (1 μg) with Superscript II (Invitrogen) in the presence of Cy3-dCTP (Amersham Pharmacia) using random hexamer oligonucleotides to prime cDNA synthesis. The reference control sample of Cy-3 labeled genomic DNA used for the glycerol minimal media experiments was prepared by mixing Cy3-labeled dCTP and BCG genomic DNA with Klenow DNA polymerase in the presence of random primers. The reaction was incubated for a minimum of 90 minutes at 37°C and the labeled DNA purified using a Qiagen MiniElute column.

The DNA microarrays provided by the Bacterial Microarray Group at St Georges (http://bugs.sgul.ac.uk/index.php) were constructed from PCR-amplified ORF-specific DNA, representing all of the predicted open reading frames from the *M. tuberculosis* H37Rv genome spotted in duplicate. The array designs are available in BμG@Sbase (Accession No. A-BUGS-1 and A-BUGS-2; http://bugs.sgul.ac.uk/A-BUGS-1) and also ArrayExpress (Accession No. A-BUGS-1 and A-BUGS-2). Pre-hybridisation, hybridisation and washing were performed as described by Stewart *et al.* [50].

Data processing and statistical analysis

Microarrays were scanned using a 428 Array Scanner (Affymetrix). Fluorescence intensity data from each array were automatically quantified using BlueFuse for microarrays (version 3.3) software (Bluegnome). The spot confidence was also evaluated by Bluefuse and low quality spots (flag E) were rejected. Duplicate spots were averaged and filtered so that only genes for which there was a value for at least 4 replicates were statistically analysed.

Microarray data were analysed by the Rank Product analysis [11] using the Bioconductor package RankProd [51] in R [52].This generates a lists of genes ranked according to log ratio and also calculates a conservative estimate of the percentage of false positives (pfp) or false discovery rate (FDR). The pfp considers the problem of multiple testing and does not require an additional correction method. The genes with PFP values smaller than 0.1 (10%) were regarded as differentially represented. Fully annotated microarray data have been deposited in BμG@Sbase (accession number E-BUGS-83; http://bugs.sgul.ac.uk/E-BUGS-83) and also ArrayExpress (accession number E-BUGS-83).

Genetic manipulations

For the construction of *mdh*, *glpK* and *mce1* mutants, *E. coli* strain DH5α was grown in solid or liquid Luria-Bertani (LB) medium as described by Sambrook *et al.* [53]. *M. bovis* BCG cells were propagated in Middlebrook 7H9 broth or 7H11 agar containing 5% (v/v) OADC enrichment media supplement (Becton Dickenson), 0.5% glycerol plus 0.05% Tween 80 for liquid cultures. For mycobacteria, when selection was required, kanamycin at 20 μg ml^{-1}, X-gal at 50 μg ml^{-1}, hygromycin at 50 g ml^{-1} and sucrose at 2% (w/v) were added to the culture media.

Mutants of *M. bovis* BCG and *M. tuberculosis* were constructed using the strategy described by Stewart *et al*, 2001. Approximately 1 kb regions flanking *mdh* and *glpK* were amplified by PCR using the Roche Expand High Fidelity PCR system. Fragments were cloned either side of the hygromycin cassette into the suicide vector pG5, which is a pSMT100 [27] plasmid carrying a kanomycin resistance gene in addition to the *sacB* counter-selectable marker. The resulting plasmid was electroporated into *M. tuberculosis* H37Rv and *M. bovis* BCG as described previously [54]. Double crossovers were selected for on 7H11 agar supplemented with kanamycin, hygromycin and X-gal. To restore the wild-type phenotype a cloned *glpK* gene was reintroduced into Δ*glpK M. bovis* BCG and Δ*glpK M. tuberculosis* on the pKinta plasmid [50]. To facilitate the inactivation of the *mdh* gene, a diploid strain was constructed by integrating a second copy of the gene at the *attB* site [50]. Deletion of the chromosomal copy of *mdh* was performed as described above. PCR and Southern analysis were performed to verify the expected genotypes.

The *mce1* operon was disrupted in *M. tuberculosis* strain H37Rv using a suicide vector kindly provided by Lee Riley and the two step counter selection cloning strategy described by Parish and Stoker [54]. This vector targets the gene Rv0168. It was previously demonstrated that Rv0168 mutants do not express any of the genes in the *mce1* operon [46]. Potential mutants were verified by PCR and Southern blot hybridization.

Supporting Information

Dataset S1 List of significant *M. bovis* BCG genes (pfp<0.1) genes uniquely required for survival in Roisin's minimal medium.
Found at: doi:10.1371/journal.pone.0005349.s001 (0.04 MB XLS)

Dataset S2 List of significant (pfp<0.1) negatively selected *M. bovis* BCG mutants at slow (td = 69 h, D = 0.01 h) growth rate.
Found at: doi:10.1371/journal.pone.0005349.s002 (0.03 MB XLS)

Dataset S3 List of significant (pfp<0.1)negatively selected *M. bovis* BCG mutants at fast (td = 23 h, D = 0.03 h) growth rate.
Found at: doi:10.1371/journal.pone.0005349.s003 (0.03 MB XLS)

Dataset S4 List of significant (pfp<0.1) *M. bovis* BCG genes required for the switch from fast to slow growth rate
Found at: doi:10.1371/journal.pone.0005349.s004 (0.03 MB XLS)

Dataset S5 List of significant (pfp<0.1) *M. bovis* BCG genes required for the switch from slow to fast growth rate.
Found at: doi:10.1371/journal.pone.0005349.s005 (0.06 MB XLS)

Dataset S6 List of significant (pfp<0.1) positively selected *M. bovis* BCG mutants at slow growth rate.
Found at: doi:10.1371/journal.pone.0005349.s006 (0.03 MB XLS)

Dataset S7 List of positively selected significant (pfp<0.1) *M. bovis* BCG mutants at fast (td = 23 h, D = 0.03 h) growth rate.
Found at: doi:10.1371/journal.pone.0005349.s007 (0.03 MB XLS)

Dataset S8 List of the positively selected significant (pfp<0.1) *M. bovis* BCG mutants during the switch from fast to slow growth rate.
Found at: doi:10.1371/journal.pone.0005349.s008 (0.03 MB XLS)

Dataset S9 List of the positively selected significant (pfp<0.1) *M. bovis* BCG mutants during the switch from slow to fast growth rate.
Found at: doi:10.1371/journal.pone.0005349.s009 (0.05 MB XLS)

Acknowledgments

The authors thank Eric Rubin for generously providing the BCG transposon library and also Lee Riley for providing us with the *mce1* operon knock out vector. We are grateful to the Wellcome Trust–funded multi-collaborative microbial pathogen microarray group at St. George's Hospital Medical School in London for providing *M. tuberculosis* microarrays.

Author Contributions

Conceived and designed the experiments: DJVB GRS. Performed the experiments: DJVB ME. Analyzed the data: DJVB BB AMK. Wrote the paper: DJVB GRS JM.

References

1. World Health Organisation (2008) Tuberculosis Fact Sheet.
2. Stewart GR, Robertson BD, Young DB (2003) Tuberculosis: a problem with persistence. Nat Rev Micro 1: 97–105.
3. Beste DJV, Peters J, Hooper T, Avignone-Rossa C, Bushell ME, et al. (2005) Compiling a Molecular Inventory for *Mycobacterium bovis* BCG at Two Growth Rates: Evidence for Growth Rate-Mediated Regulation of Ribosome Biosynthesis and Lipid Metabolism. J Bacteriol 187: 1677–1684.
4. Beste DJV, Laing E, Bonde B, Avignone-Rossa C, Bushell ME, et al. (2007) Transcriptomic Analysis Identifies Growth Rate Modulation as a Component of the Adaptation of Mycobacteria to Survival inside the Macrophage. J Bacteriol 189: 3969–3976.
5. Garton NJ, Waddell SJ, Sherratt AL, Lee SM, Smith RJ, et al. (2008) Cytological and Transcript Analyses Reveal Fat and Lazy Persister-Like Bacilli in Tuberculous Sputum. PLoS Medicine 5: e75.

6. Kendall SL, Rison SCG, Movahedzadeh F, Frita R, Stoker NG (2004) What do microarrays really tell us about *M. tuberculosis*? Trends in Microbiology 12: 537–544.
7. Rengarajan J, Bloom BR, Rubin EJ (2005) Genome-wide requirements for *Mycobacterium tuberculosis* adaptation and survival in macrophages. PNAS 102: 8327–8332.
8. Sassetti CM, Boyd DH, Rubin EJ (2003) Genes required for mycobacterial growth defined by high density mutagenesis. Molecular Microbiology 48: 77–84.
9. Chan K, Kim CC, Falkow S (2005) Microarray-Based Detection of *Salmonella enterica* Serovar Typhimurium Transposon Mutants That Cannot Survive in Macrophages and Mice. Infect Immun 73: 5438–5449.
10. Sassetti CM, Rubin EJ (2003) Genetic requirements for mycobacterial survival during infection. Proceedings of the National Academy of Sciences of the United States of America 100: 12989–12994.
11. Breitling R, Armengaud P, Amtmann A, Herzyk P (2004) Rank products: a simple, yet powerful, new method to detect differentially regulated genes in replicated microarray experiments. Febs Letters 573: 83–92.
12. Hong F, Breitling R (2008) A comparison of meta-analysis methods for detecting differentially expressed genes in microarray experiments. Bioinformatics 24: 374–382.
13. Rodriguez GM, Voskuil MI, Gold B, Schoolnik GK, Smith I (2002) ideR, An essential gene in *Mycobacterium tuberculosis*: role of IdeR in iron-dependent gene expression, iron metabolism, and oxidative stress response. Infect Immun 70: 3371–3381.
14. Domenech P, Reed MB, Barry CE III (2005) Contribution of the *Mycobacterium tuberculosis* MmpL Protein Family to Virulence and Drug Resistance. Infect Immun 73: 3492–3501.
15. Keating LA, Wheeler PR, Mansoor H, Inwald JK, Dale J, et al. (2005) The pyruvate requirement of some members of the *Mycobacterium tuberculosis* complex is due to an inactive pyruvate kinase: implications for in vivo growth. Molecular Microbiology 56: 163–174.
16. Lin ECC (1976) Glycerol Dissimilation and its Regulation in Bacteria. Annual Review of Microbiology 30: 535–578.
17. Goldman DS (1963) ENZYME SYSTEMS IN THE MYCOBACTERIA XV.: Initial Steps in the Metabolism of Glycerol. J Bacteriol 86: 30–37.
18. De Voss JJ, Rutter K, Schroeder BG, Su H, Zhu Y, et al. (2000) The salicylate-derived mycobactin siderophores of *Mycobacterium tuberculosis* are essential for growth in macrophages. Proceedings of the National Academy of Sciences of the United States of America 97: 1252–1257.
19. Beste D, Hooper T, Stewart G, Bonde B, Avignone-Rossa, et al. (2007) GSMN-TB: a web-based genome-scale network model of *Mycobacterium tuberculosis* metabolism. Genome Biology 8: R89.
20. Movahedzadeh F, Rison SCG, Wheeler PR, Kendall SL, Larson TJ, et al. (2004) The *Mycobacterium tuberculosis* Rv1099c gene encodes a GlpX-like class II fructose 1,6-bisphosphatase. Microbiology 150: 3499–3505.
21. Gu X, Chen M, Shen H, Jiang X, Huang Y, et al. (2006) Rv2131c gene product: An unconventional enzyme that is both inositol monophosphatase and fructose-1,6-bisphosphatase. Biochemical and Biophysical Research Communications 339: 897–904.
22. Rickman L, Saldanha JW, Hunt DM, Hoar DN, Colston MJ, et al. (2004) A two-component signal transduction system with a PAS domain-containing sensor is required for virulence of *Mycobacterium tuberculosis* in mice. Biochemical and Biophysical Research Communications 314: 259–267.
23. Glover RT, Kriakov J, Garforth SJ, Baughn AD, Jacobs WR Jr (2007) The Two-Component Regulatory System senX3-regX3 Regulates Phosphate-Dependent Gene Expression in *Mycobacterium smegmatis*. J Bacteriol 189: 5495–5503.
24. Pandey DP, Gerdes K (2005) Toxin-antitoxin loci are highly abundant in free-living but lost from host-associated prokaryotes. Nucl Acids Res 33: 966–976.
25. Camacho LR, Constant P, Raynaud C, Laneelle MA, Triccas JA, et al. (2001) Analysis of the Phthiocerol Dimycocerosate Locus of *Mycobacterium tuberculosis*. Evidence that this lipid is involved in the cell wall permeability barrier. J Biol Chem 276: 19845–19854.
26. Rousseau C, Winter N, Pivert E, Bordat Y, Neyrolles O, et al. (2004) Production of phthiocerol dimycocerosates protects *Mycobacterium tuberculosis* from the cidal activity of reactive nitrogen intermediates produced by macrophages and modulates the early immune response to infection. Cellular Microbiology 6: 277–287.
27. Stewart GR, Snewin VA, Walzl G, Hussell T, Tormay P, et al. (2001) Overexpression of heat-shock proteins reduces survival of *Mycobacterium tuberculosis* in the chronic phase of infection. Nat Med 7: 732–737.
28. Chen P, Ruiz RE, Li Q, Silver RF, Bishai WR (2000) Construction and characterization of a *Mycobacterium tuberculosis* mutant lacking the alternate sigma factor gene, sigF. Infect Immun 68: 5575–5580.
29. Hu Y, Kendall S, Stoker NG, Coates ARM (2004) The Mycobacterium tuberculosis *sigJ* gene controls sensitivity of the bacterium to hydrogen peroxide. FEMS Microbiology Letters 237: 415–423.
30. Karls RK, Guarner J, McMurray DN, Birkness KA, Quinn FD (2006) Examination of *Mycobacterium tuberculosis* sigma factor mutants using low-dose aerosol infection of guinea pigs suggests a role for SigC in pathogenesis. Microbiology 152: 1591–1600.
31. Geiman DE, Kaushal D, Ko C, Tyagi S, Manabe YC, et al. (2004) Attenuation of late-stage disease in mice infected by the *Mycobacterium tuberculosis* mutant lacking the SigF alternate sigma factor and identification of SigF-dependent genes by microarray analysis. Infect Immun 72: 1733–1745.
32. Williams EP, Lee JH, Bishai WR, Colantuoni C, Karakousis PC (2007) *Mycobacterium tuberculosis* SigF Regulates Genes Encoding Cell Wall-Associated Proteins and Directly Regulates the Transcriptional Regulatory Gene phoY1. J Bacteriol 189: 4234–4242.
33. Downing KJ, Betts JC, Young DI, McAdam RA, Kelly F, et al. (2004) Global expression profiling of strains harbouring null mutations reveals that the five rpf-like genes of *Mycobacterium tuberculosis* show functional redundancy. Tuberculosis 84: 167–179.
34. Kana BD, Ghordhan BG, Downing KJ, Sung N, Vostroknutova G, et al. (2008) The resuscitation-promoting factors of *Mycobacterium tuberculosis* are required for virulence and resuscitation from dormancy but are collectively dispensable for growth in vitro. Mol Microbiol 67: 672–684.
35. Hett EC, Chao MC, Steyn AJ, Fortune SM, Deng LL, et al. (2007) A partner for the resuscitation-promoting factors of *Mycobacterium tuberculosis*. Mol Microbiol 66: 658–668.
36. Zahrt TC, Deretic V (2001) *Mycobacterium tuberculosis* signal transduction system required for persistent infections. Proc Natl Acad Sci U S A 98: 12706–12711.
37. Pang X, Vu P, Byrd TF, Ghanny S, Soteropoulos P, et al. (2007) Evidence for complex interactions of stress-associated regulons in an mprAB deletion mutant of Mycobacterium tuberculosis. Microbiology 153: 1229–1242.
38. Joshi SM, Pandey AK, Capite N, Fortune SM, Rubin EJ, et al. (2006) Characterization of mycobacterial virulence genes through genetic interaction mapping. PNAS 103: 11760–11765.
39. Stewart GR, Patel J, Robertson BD, Rae A, Young DB (2005) Mycobacterial mutants with defective control of phagosomal acidification. PLoS Pathog 1: e33.
40. Baganz FA, Hayes A, Farquhar R, Butler PR, Gardner DCJ, et al. (1999) Quantitative analysis of yeast gene function using competition experiments in continuous culture. Yeast 14: 1417–1427.
41. El Hajji M, Rapaport A (2009) Practical coexistence of two species in the chemostat - A slow-fast characterization. Mathematical Biosciences 218: 33–39.
42. Lobry C, Harmand J (2006) A new hypothesis to explain the coexistence of n species in the presence of a single resource. Comptes Rendus Biologies 329: 40–46.
43. Arruda S, Bomfim G, Knights R, Huima-Byron T, Riley LW (1993) Cloning of an *M. tuberculosis* DNA fragment associated with entry and survival inside cells. Science 261: 1454–1457.
44. Cole ST, Brosch R, parkhill T, Garnier T, Churcher D, et al. (1998) Deciphering the biology of *Mycobacterium tuberculosis* from the complete genome sequence. Nature 393: 537–544.
45. Pandey AK, Sassetti CM (2008) Mycobacterial persistence requires the utilization of host cholesterol. PNAS 105: 4376–4380.
46. Shimono N, Morici L, Casali N, Cantrell S, Sidders B, et al. (2003) Hypervirulent mutant of *Mycobacterium tuberculosis* resulting from disruption of the mce1 operon. Proceedings of the National Academy of Sciences of the United States of America 100: 15918–15923.
47. Lima P, Sidders B, Morici L, Reader R, Senaratne R, et al. (2007) Enhanced mortality despite control of lung infection in mice aerogenically infected with a *Mycobacterium tuberculosis* mce1 operon mutant. Microbes and Infection 9: 1285–1290.
48. Vulic M, Kolter R (2001) Evolutionary Cheating in *Escherichia coli* Stationary Phase Cultures. Genetics 158: 519–526.
49. Uchida Y, Casali N, White A, Morici L, Kendall LV, et al. (2007) Accelerated immunopathological response of mice infected with *Mycobacterium tuberculosis* disrupted in the mce1 operon negative transcriptional regulator. Cell Microbiol 9: 1275–1283.
50. Stewart GR, Wernisch L, Stabler R, Mangan JA, Hinds J, et al. (2002) Dissection of the heat-shock response in *Mycobacterium tuberculosis* using mutants and microarrays. Microbiology 148: 3129–3138.
51. Hong F, Breitling R, McEntee CW, Wittner BS, Nemhauser JL, et al. (2006) RankProd: a bioconductor package for detecting differentially expressed genes in meta-analysis. Bioinformatics 22: 2825–2827.
52. R Development Core Team (2006) R: A language and environment for statistical computing, R Foundation for Statistical Computing. Vienna, Austria.
53. Sambrook J, Fritsch EF, Maniatis T (1989) Molecular Cloning: A Laboratory Manual. Cold Spring Harbor, New York: Cold Spring Harbor Laboratory Press.
54. Parish T, Stoker NG (2000) Use of a flexible cassette method to generate a double unmarked *Mycobacterium tuberculosis* tlyA plcABC mutant by gene replacement. Microbiology 146(Pt 8): 1969–1975.
