# Supplementary material for: A systematic evaluation of Mycobacterium tuberculosis Genome-Scale Metabolic Networks
Source: PLoS Comput Biol. 2020 Jun 15;16(6):e1007533. doi: 10.1371/journal.pcbi.1007533 (PMC7316355; doi:10.1371/journal.pcbi.1007533)
Supplement: S8 File — (ZIP) [file pcbi.1007533.s040.zip › Cholesterol_Essentiality(Griffin_data)/medium/Griffin_MinimalMedium.docx]

**Griffin et al., 2011 Minimal Medium:** Asparagine, PO4, K, H, Na, Fe2, Fe3, NH4+, Citrate, SO4, Mg, Ca, Cl2, Zn, Glycerol, ethanol

**Griffin et al.,2011 Minimal Medium Cholesterol** For cholesterol experiments bacteria were grown in minimal media containing asparagine 0.5g/L, KH_2_PO_4_ 1.0g/L, Na_2_HPO_4_ 2.5g/L, ferric ammonium citrate 50mg/L, MgSO_4_ ·7H_2_0 0.5g/L, CaCl_2_ 0.5g/L, ZnSO_4_ 0.1mg/L), 0.2% tyloxapol, 0.2% ethanol and either 0.1% glycerol or 0.01% cholesterol….
