# Supplementary material for: A systematic evaluation of Mycobacterium tuberculosis Genome-Scale Metabolic Networks
Source: PLoS Comput Biol. 2020 Jun 15;16(6):e1007533. doi: 10.1371/journal.pcbi.1007533 (PMC7316355; doi:10.1371/journal.pcbi.1007533)
Supplement: Attachment — Submitted filename: Beste_Victor_rebuttal_april2020.docx [file pcbi.1007533.s041.docx]

Dear Dr Anders Wallqvist,

Thank you for the comments and recommendations regarding our manuscript entitled: A systematic evaluation of Mycobacterium tuberculosis Genome-Scale Metabolic Networks.

We are very happy that the reviewers recognised the significance of the study and believe that “this study adds significant value to the TB research community” and a “welcome analysis in the literature, given the growing number of models and derivations of models and the need for reconciliation and assessment of the current state”.

We have carefully considered all the comments and have prepared a revised manuscript, as you requested. Please find below a detailed account of all changes introduced into the manuscript and detailed response to the concerns raised by the Referees.

We hope that you will now find our manuscript acceptable for publication in PLoS Computational Biology.

Yours Sincerely

Dany Beste

Reviewer's Responses to Questions

Reviewer #1:

1. It is highly recommended by the COBRA community that the authors provide a MEMOTE report of both the compared and improved reconstructions (https://www.biorxiv.org/content/10.1101/350991v1). Rather than dictating whether the models are worthy of being published, the MEMOTE report should help understand what the limitations are and where they should focus their efforts in potentially improving the model. Importantly, the MEMOTE report may help the authors improve the refined models and comparisons before publication.

Both sMtb2.0 and iEK1011_2.0 were run in memote. The memote report is attached in S3 File.

We have included the following paragraph in “Refining Mtb GSMNs” section: P23, L429-434. This text is highlighted in the modified manuscript

2. The authors should additionally provide the improved reconstructions in both json and sbml format.

We now include sMtb2.0 and iEK1012_2.0 GSMNs in json and sbml formats in file S5.

The authors should add another section detailing the different biomass reactions across the models (i.e., metabolite composition, metabolite weightings, shadow prices, etc). Currently, only a comparison of the biomass molecular weight is provided at the end of the “Checking mass and charge balances of biochemical reactions” section.

We have now included a section on the biomass formulation (P7, L147-157). This text is highlighted in the modified manuscript

3. The authors should provide a description/comparison of the growth-associated maintenance (GAM) or non-growth associated maintenance (NGAM) reactions across the different TB models. One or two sentences is sufficient.

This is now included (P8, L159-171)

4. Both flux variability analysis (FVA) and flux sampling are key FBA tools used to evaluate the solution space of GEMs. We recommend that the authors perform FVA and flux sampling of the improved GEMs (iEK1011_2.0 and sMtb2.0) to both evaluate the flux bottlenecks and distributions between models.

We have now performed pathway utilization analysis using Flux variability analysis and flux sampling for the improved models focusing on central carbon metabolism and extracellular reactions. The results are described in a new section named “Pathway utilization of sMtb2.0 and iEK1011_2.0” P23 L435-479.

5. Continuing from comment 5, the authors should provide detailed comparisons of the flux profile between the improved GEMs (iEK1011_2.0 and sMtb2.0) in standard TB media using either parsimonious FBA (pFBA), FVA, or flux sampling. For example, do the models prefer different nutrients? Do they have similar flux strategies in TCA and oxPPP? We would like to see the authors detail differences in pathway utilization strategies between the improved models, if there are any.

P23, L435-479. we have now performed comparisons of the flux profiles obtained using the improved GEMS growing on Roisin’s minimal media.

Minor comments

1. Line 100: The description of red in Fig 2 is written the same as blue.

This has now been changed

Reviewer #2:

Major concerns

• The paragraph starting on line 38 is not sufficient to describe the examined models and how they differ. The order of publication and combination of models is included in figure 1 and further detail is provided in S1 but there is no mention of it in the main text.

We have now included a brief introduction to the other models depicted in Figure 1. This can be found on P2-3, L47-70

• The model with the greatest number of reactions and genes is iAB-AMF-1410-Mt-661 updated published on 2017. Why was this model ignored? There is no mention of this model. How can the purported updated models be considered most complete if they still have fewer reactions and genes than the noted model?

The iAB-AMF-1410-Mt-661 is a host-pathogen integrated genome-scale model that combines the metabolic reactions of human macrophages and Mtb inside a phagosome compartment and therefore it is obviously significantly larger as it contains reactions for macrophage metabolism (represented by Recon 1 [1]) and Mtb metabolism (represented by iNJ661 [2]). Our goal was to analyse GSMN-Mtb models in order to guide researchers for future systems biology applications, therefore host-pathogen integrations like iAB-AMF-1410-Mt-661 were not analyzed in this manuscript.

• Examining figure 2 it is obvious that some models are just the old models plus some new reactions e.g. sMtb and sMtb2018. Why include the former when instead iAB-AMF-1410-Mt-661 could have been examined?

As already discussed above the iAB-AMF-1410-Mt-661 is a hybrid model for capturing host-pathogen interactions.

• Line 63, what do you mean by multi-scale simulation platform? What scales are you talking about?

We have replaced this statement in P3, L82-84 with

“In addition, we present updated versions of the highest performing models iEK1011 and sMtb2018 (iEK1011_2.0 and sMtb2.0) for the TB research community to use to study the metabolism of this deadly pathogen.”

• Line 92, I’m confused about the statement “has poor annotation of genes”? Does that mean the genes identified were incorrectly assigned a function or that the coverage of the pathways was lacking?

We have clarified this statement by changing the text in P5, L111-113 to the below

“Likewise, the iNJ661v_modified model (Fig 1) has an insufficient number of genes involved in lipid-metabolism (e.g. β-oxidation, cholesterol degradation, fatty acid biosynthesis, lipid biosynthesis and mycolic acid biosynthesis).

• How does iCG760 operate as a system-level model without water as one of its metabolites? To me the sorry biochemical states of three of the published models is the most shocking find of the paper.

GSMNs can operate without water or any currency metabolites in their stoichiometric matrices, but their flux predictions can be negatively affected. Water and protons should of course be added in both sides of the biochemical reactions to satisfy charge and mass balances of biochemical reactions and improve the accuracy of fluxes predictions [3]. Appropriate text has been added to emphasise this point P19, L135-136.

• When highlighting the results of analyses for blocked reactions and dead-end metabolites it is important to note if the cause is due to gaps in genome or incomplete GPRs. The former could be viewed as the modeler trying not to add a new phenotype without experimental validation whereas the latter is a poorly curated model.

This is an interesting suggestion for checking the quality of reconstruction. However, doing a detailed track for identifying the cause of all gaps in the eight Mtb GSMNs is a formidable effort that is beyond the scope of this work.

• Why not combine sMtb2.0 and iEK1011_2.0 into one unified model that would work well with Recon for host pathogen interactions as well as correctly predicts growth on a variety of different compounds? That would be the perfect outcome from your extensive efforts.

Due to different nomenclature of the two models this is actually a formidable task which is beyond the scope of this work and actually we don’t think this is the right approach. Led by experience from the yeast [4,5] and Escherichia coli [6–9] which have a longer history of genome scale metabolic reconstructions, sometimes different models may be preferable for use in different applications [10–15]. And actually, the largest model is not necessarily the most accurate.

Minor concerns

• Do not use acronyms in the abstract prior to defining them, i.e. TB

Corrected

• Some figures are labeled Fig. others Figure. Please choose one format.

Corrected

• The sentence starting on line 152 is incomplete.

This has been corrected in P10, L198-201 to: “Of those models that contained a pathway for cholesterol degradation (GSMN-TB1.1, iCG760, iSM810, iEK1011, sMtb, and sMtb2018) the GSMN-TB1.1 cholesterol degrading pathway contained a number of dead end metabolites making this model unsuitable for exploring the metabolism of this important in vivo carbon source. ”

Reviewer #3: The article entitled, 'A systematic evaluation of Mycobacterium tuberculosis Genome-Scale Metabolic Networks', Lopez-Agudelo et al present a comparative analysis of Mtb genome scale metabolic reconstructions.

1. How did the authors arrive at the cutoff of 10% for the deviation of 1 g/mmol? Why is iEK1011 so much higher than some of the other models?

This isn’t a cut-off value. We just found that the Molecular Weight deviation of the iEK1011 model is 9%, the “10%” was used for highlighting that deviations in all the Mtb models are below 10%. The deviation of biomass molecular weight in iEK1011 is due to the biomass reaction, which includes components from sMtb and the iOSDD890 biomass reactions.

We have now included in the text (P9, L180-181) “The higher value obtained for iEK1011 is because the biomass formulation is a hybrid of sMtb and the iOSDD890 biomass reactions (S8 Table).” to clarify this.

2. It is understandable to revise reaction directionality based on thermodynamic feasibility assessment, however to remove a particular reaction(s) (DFRA1 and DFRA3) should require a little more justification, including any experimental support/arguments and/or explanations of why the arguments made for a different genus (yeast) are also relevant for Mtb.

A major TIC in sMtb2.0 contain parallel reactions such as Dihidrofolate reductases (DFRA 1, DFRA2, DFRA3, and DFRA4). For instance, DFRA 1 and DFRA 2, and DFRA 3 and DFRA4 are parallel reactions that catalyze the same reaction but with a different currency metabolite as NADH/NADPH. Pereira and colleagues demonstrate that by enforcing the cofactors use for NADPH/NADP in anabolic reactions and NADH/NAD+ for catabolic reactions allows more accurate flux distributions [16]. Biosynthesis of cofactors is considered anabolic metabolism, therefore, we decided to eliminate DFRA 1 and DFRA 3 from the model.

We have clarified this statement in P20, L396-401.

3. the identification of a need for a menaquinone-dependent succinate dehydrogenase reaction is an interesting modification that appeared to improve the predictive capability of the model. However the authors state that they had to add this reaction to iEK1011, whereas in reality all that was required was to change this from an irreversible to a reversible reaction. It would be helpful to note whether there is experimental evidence to suggest that concentrations are present to enable thermodynamic reversibility of the reaction. Additionally, will the reversible form of the reaction result in any infeasible reaction cycles?

The iEK1011 model contains only one succinate dehydrogenase reaction named SUCD1 (succ_c[c] + fad_c[c] => fum_c[c] + fadh2_c[c]). However, this reaction is not coupled directly with electron transport chain by coupling the succinate oxidation to fumarate with the reduction of menaquinone electron carriers [2,3]. The reaction we added to iEK1011_2.0 was the irreversible SDH1 reaction (succ[c] + mqn8[c] => fum[c] + mql8[c]), using as cofactors menaquinone/menaquinol, which couples directly the TCA cycle with electron transport chain and oxidative phosphorylation.

Fumarate reductase and succinate dehydrogenase perform the reversible oxidation of succinate to fumarate, in GSMNs is recommended to have them in separated reactions for better description of Gene-Protein-Associations due to the existence of multiple genes annotated with these functions [17].

4. similar to the question raised on p13, which one of these involved addition of new reactions vs making reactions reversible

The irreversible reaction SDH1 was added into the iEK1011 model as an improvement to iEK1011_2.0.

+ p18: 'thermodynamically unstable' -- perhaps the authors meant thermodynamically infeasible, since there does not appear to be any discussion of stability of particular flux states

Thanks for pointing this out. This has been changed to thermodynamically infeasible

+ p17: what specifically is meant by "unblocking B12 synthesis"? The authors themselves cite specific articles that highlight the incomplete knowledge about B12 handling in Mtb.

The Mtb genome is annotated to contain all the enzymes for the synthesis of Vitamin B12 [18,19]. However, to date there is not experimental evidence that Mtb can synthetize Vitamin B12[19,20]. Under this uncertainty, we completed the Vitamin B12 pathway.

We have clarified in the manuscript by including on P19, L371-375

+ p17: it is not clear what addition is made by addition of biotin?+ p17: again, it is not clear what specifically the authors mean by "including ... pyridoxal-5-phosphate", although from the referenced article, in this case they presumably are referring to the addition of pyridoxal 5' synthase. For biotin, it is not clear what revisions they made or the rational/justification for it.

Xavier and colleagues demonstrated that addition of universal cofactors like biotin, pyridoxal-phosphate, tetrahydrofolate, among others in the biomass objective function can improve the gene essentiality predictions of an Mtb genome-scale model [8]. Therefore, we modified the objective functions of sMtb2.0 and iEK1011_2.0 by adding these cofactors including biotin and pyridoxal phosphate with the same stoichiometric coefficients used by Xavier and Colleagues. These objective functions were named “BiomassGrowth_2.0” and “BIOMASS__2.1”.

We clarified this in the text P19, L378-386

+ Table 5: it is not clear where the cited values for O2, NADH/NAD, and NADPH/NADP are obtained from the referenced citations, since the paper and textbook (non-specific reference) do not appear to cite measurements for Mtb. Additionally it is not clear what is meant by "Other metabolites".

In the absence of data for the concentration of all metabolites inside Mtb, for our NExT implementation, we decided to assume that all metabolites are in range between 0.0001 mM and 10 mM, which represents a range of observed physiological concentrations [21,22].

We clarify this in the text (P29, L577-580) by including: “In the absence of metabolite concentration data from inside Mtb, for our NExT implementation, we assumed that all metabolites are between 0.0001 mM and 10 mM, which represents a range of observed physiological concentrations used by Martinez et al [94,95].”

+ p26: What was the scientific rationale/calculation to identify the 5% cutoff for

Traditionally in silico gene essentiality analysis employs an arbitrary user-defined threshold for classifying genes as essential and non-essential [23–25]. For example, Rienksma and Colleagues, Xavier and Colleagues and Kavvas and Colleagues used growth rate thresholds of 5%, 10% and 20%, respectively, for classifying essential or non-essential genes. In our study, we defined a gene as essential if deleting it from the model reduced the in silico growth rate by at least 95%.

+ minor comments:

there are a number of small grammatical errors, e.g.

p13, l255: "is as a result"

Corrected

p16, l312: "exempler", did you mean exemplar?

Corrected

p 29: supplemental file legends: S10, S11, S12, S13, "for classify essential"

etc.

Corrected

**References:**

1.	Duarte NC, Becker SA, Jamshidi N, Thiele I, Mo ML, Vo TD, et al. Global reconstruction of the human metabolic network based on genomic and bibliomic data. Proc Natl Acad Sci U S A. 2007;104: 1777–1782. doi:10.1073/pnas.0610772104

2.	Jamshidi N, Palsson BØ. Investigating the metabolic capabilities of Mycobacterium tuberculosis H37Rv using the in silico strain iNJ 661 and proposing alternative drug targets. BMC Syst Biol. 2007;1: 26.

3.	Thiele I, Palsson BØ. A protocol for generating a high-quality genome-scale metabolic reconstruction. Nat Protoc. 2010;5: 93.

4.	Lu H, Li F, Sánchez BJ, Zhu Z, Li G, Domenzain I, et al. A consensus S. cerevisiae metabolic model Yeast8 and its ecosystem for comprehensively probing cellular metabolism. Nat Commun. 2019;10: 1–13. doi:10.1038/s41467-019-11581-3

5.	Lopes H, Rocha I. Genome-scale modeling of yeast: chronology, applications and critical perspectives. FEMS Yeast Res. 2017;17. doi:10.1093/femsyr/fox050

6.	Feist AM, Henry CS, Reed JL, Krummenacker M, Joyce AR, Karp PD, et al. A genome-scale metabolic reconstruction for Escherichia coli K-12 MG1655 that accounts for 1260 ORFs and thermodynamic information. Mol Syst Biol. 2007;3. doi:10.1038/msb4100155

7.	Reed JL, Vo TD, Schilling CH, Palsson BO. An expanded genome-scale model of Escherichia coli K-12 (iJR904 GSM/GPR). Genome Biol. 2003;4: R54. doi:10.1186/gb-2003-4-9-r54

8.	Orth JD, Conrad TM, Na J, Lerman JA, Nam H, Feist AM, et al. A comprehensive genome-scale reconstruction of Escherichia coli metabolism-2011. Mol Syst Biol. 2011;7: 535–535. doi:10.1038/msb.2011.65

9.	Monk JM, Lloyd CJ, Brunk E, Mih N, Sastry A, King Z, et al. iML1515, a knowledgebase that computes Escherichia coli traits. Nat Biotechnol. 2017;35: 904–908. doi:10.1038/nbt.3956

10.	Pandey V, Hadadi N, Hatzimanikatis V. Enhanced flux prediction by integrating relative expression and relative metabolite abundance into thermodynamically consistent metabolic models. PLoS Comput Biol. 2019;15: e1007036. doi:10.1371/journal.pcbi.1007036

11.	Mienda BS, Shamsir MS, Illias RM. Model-guided metabolic gene knockout of gnd for enhanced succinate production in Escherichia coli from glucose and glycerol substrates. Comput Biol Chem. 2016;61: 130–137. doi:10.1016/j.compbiolchem.2016.01.013

12.	Long CP, Antoniewicz MR. Metabolic flux responses to deletion of 20 core enzymes reveal flexibility and limits of E. coli metabolism. Metab Eng. 2019;55: 249–257. doi:10.1016/j.ymben.2019.08.003

13.	Zeng H, Yang A. Flux Balance Analysis Incorporating a Coarse-grained Proteome Constraint for Predicting Overflow Metabolism in Escherichia Coli. Computer Aided Chemical Engineering. Elsevier; 2019. pp. 865–870. doi:10.1016/B978-0-12-818634-3.50145-4

14.	Wayman JA, Glasscock C, Mansell TJ, DeLisa MP, Varner JD. Improving designer glycan production in Escherichia coli through model-guided metabolic engineering. Metab Eng Commun. 2019;9: e00088. doi:10.1016/j.mec.2019.e00088

15.	Mesquita TJB, Sargo CR, Fuzer JR, Paredes SAH, Giordano RDC, Horta ACL, et al. Metabolic fluxes-oriented control of bioreactors: A novel approach to tune micro-Aeration and substrate feeding in fermentations. Microb Cell Fact. 2019;18: 150. doi:10.1186/s12934-019-1198-6

16.	Pereira R, Nielsen J, Rocha I. Improving the flux distributions simulated with genome-scale metabolic models of Saccharomyces cerevisiae. Metab Eng Commun. 2016;3: 153–163. doi:10.1016/j.meteno.2016.05.002

17.	Cook GM, Hards K, Vilchèze C, Hartman T, Berney M. Energetics of Respiration and Oxidative Phosphorylation in Mycobacteria. Microbiol Spectr. 2014;2. doi:10.1128/microbiolspec.mgm2-0015-2013

18.	Gopinath K, Moosa A, Mizrahi V, Warner DF. Vitamin B12 metabolism in Mycobacterium tuberculosis. Future Microbiol. 2013;8: 1405–1418. doi:10.2217/fmb.13.113

19.	Young DB, Comas I, de Carvalho LPS. Phylogenetic analysis of vitamin B12-related metabolism in Mycobacterium tuberculosis. Front Mol Biosci. 2015;2: 1–14. doi:10.3389/fmolb.2015.00006

20.	Minias A, Minias P, Czubat B, Dziadek J. Purifying selective pressure suggests the functionality of a vitamin B12 biosynthesis pathway in a global population of mycobacterium tuberculosis. Genome Biol Evol. 2018;10: 2326–2337. doi:10.1093/gbe/evy153

21.	Martínez VS, Quek LE, Nielsen LK. Network thermodynamic curation of human and yeast genome-scale metabolic models. Biophys J. 2014;107: 493–503. doi:10.1016/j.bpj.2014.05.029

22.	Martínez VS, Nielsen LK. NExT: Integration of thermodynamic constraints and metabolomics data into a metabolic network. Methods in Molecular Biology. Springer; 2014. pp. 65–78. doi:10.1007/978-1-4939-1170-7_4

23.	Rienksma RA, Suarez-Diez M, Spina L, Schaap PJ, dos Santos VAPM. Systems-level modeling of mycobacterial metabolism for the identification of new (multi-) drug targets. Seminars in immunology. Elsevier; 2014. pp. 610–622.

24.	Xavier JC, Patil KR, Rocha I. Integration of biomass formulations of genome-scale metabolic models with experimental data reveals universally essential cofactors in prokaryotes. Metab Eng. 2017;39: 200–208.

25.	Kavvas ES, Seif Y, Yurkovich JT, Norsigian C, Poudel S, Greenwald WW, et al. Updated and standardized genome-scale reconstruction of Mycobacterium tuberculosis H37Rv, iEK1011, simulates flux states indicative of physiological conditions. BMC Syst Biol. 2018;12: 25.
